# Exploring community and expert perceptions of the acceptability of an oropharyngeal gonorrhoea controlled human infection model in Australia

**DOI:** 10.1038/s41598-025-30975-6

**Published:** 2025-12-02

**Authors:** Eloise Williams, J. E. Moulton, E. Jamrozik, J. Prestedge, S. Ruth, C. K. Fairley, M. Y. Chen, D. A. Williamson, J. S. McCarthy, J. S. Hocking, L. Keogh

**Affiliations:** 1 Department of Infectious Diseases, The University of Melbourne at the Peter Doherty Institute for Infection and Immunity, Melbourne, VIC Australia; 2 Victorian Infectious Diseases Reference Laboratory, Royal Melbourne Hospital at the Peter Doherty Institute for Infection and Immunity, Melbourne, VIC Australia; 3 Melbourne School of Population and Global Health, The University of Melbourne, Melbourne, VIC Australia; 4 Ethox and Pandemic Sciences Institute, University of Oxford, Oxford, UK; 5 The Walter and Eliza Hall Institute of Medical Research, Melbourne, VIC Australia; 6 Thorne Harbour Health, Melbourne, VIC Australia; 7 Melbourne Sexual Health Centre, Alfred Health, Melbourne, VIC Australia; 8 Central Clinical School, Monash University, Melbourne, VIC Australia; 9 School of Medicine, University of St Andrews, Fife, Scotland; 10 Victorian Infectious Diseases Service, The Royal Melbourne Hospital at the Peter Doherty Institute for Infection and Immunity, Melbourne, VIC Australia

**Keywords:** Diseases, Health care, Medical research

## Abstract

The development of an oropharyngeal gonorrhoea controlled human infection model (CHIM) could result in important translational outcomes, including accelerated development of new drugs and vaccines. Ethical study design for such a model requires community consultation, including assessment of the acceptability of the proposed CHIM among key stakeholders. This qualitative study involved: (i) semi-structured interviews and focus groups with individuals who would be eligible for participation in the proposed oropharyngeal gonorrhoea CHIM, defined as healthy men who have sex with men (MSM) aged 18–50 years living in Victoria (Australia); and, (ii) semi-structured interviews with gonorrhoea experts. Data were analysed using inductive thematic analysis supported by NVivo. Twenty-seven semi-structured interviews and one focus group were undertaken with 32 individuals between July and November, 2024, comprising 22 potential CHIM participants, and 10 experts. Overall, an oropharyngeal gonorrhoea CHIM was acceptable to most participants. Financial compensation and including only MSM who do not have sex with women were highlighted as key areas of debate. Participants highlighted that recruitment strategies should be sensitive to the stigma associated with gonorrhoea and history of stigma experienced by MSM. An oropharyngeal gonorrhoea CHIM is acceptable to key stakeholders but must be carefully designed to avoid exacerbation of stigma.

**Supplementary Information:**

The online version contains supplementary material available at 10.1038/s41598-025-30975-6.

## Introduction

*Neisseria gonorrhoeae*, the bacteria that causes the sexually transmitted infection (STI) gonorrhoea, was estimated to cause 88 million infections in 2019, with increasing incidence observed worldwide^1^. Gonorrhoea is a highly stigmatized infection^2^ that disproportionately affects marginalised populations who may already experience stigma and discrimination, including people from low- and middle-income countries, people living with HIV (PLHIV), men who have sex with men (MSM), sex workers and indigenous populations^3^. The primary syndrome caused by *N. gonorrhoeae* is urogenital gonorrhoea^4^ which may cause long-term harm for people with a uterus^5^. Asymptomatic infection is the predominant manifestation of *N. gonorrhoeae* infection at extragenital sites such as the oropharynx^6^.

The oropharynx has been identified as an important anatomical site of infection given its potential role in gonorrhoea transmission^7^ and the development of antimicrobial resistance (AMR)^8^. *N. gonorrhoeae* AMR and the evolution of strains of the pathogen that are highly resistant to recommended antibiotics such as ceftriaxone, has been identified as a critical threat to public health^9,10^. As such, *N. gonorrhoeae* has been classified as a high priority pathogen for development of new antibiotics and vaccines by the World Health Organization (WHO)^11,12^.

Controlled human infection models (CHIMs) represent a specialised form of clinical trial that entails intentional exposure of consenting volunteers to a pathogen, with the aim of obtaining scientific data about the host-pathogen interaction or for testing of interventions^13^. CHIMs are increasingly recognized as key platforms for the detailed study of infectious disease pathogenesis and for preliminary assessment of new drugs and vaccines^14^. In general, participants in CHIMs are monitored carefully during the period of experimental infection with clinical history, examination and laboratory sampling. Participants are then provided with curative antimicrobial treatment after a defined period of experimental infection (usually 5–14 days) or if earlier if they develop pre-defined symptoms that may signal more severe infection.

A gonorrhoea penile urethritis CHIM has been used for pathogenesis studies in the United States since the 1980s with no serious adverse events reported^15^. An oropharyngeal gonorrhoea CHIM has not previously been performed. As oropharyngeal *N. gonorrhoeae* causes a readily treatable infection and/or disease, affects an anatomical site that is readily sampled, and results in no irreversible pathology, an outpatient oropharyngeal gonorrhoea CHIM offers an attractive opportunity for translational research with minimal risks to participants, thus having the potential to accelerate the development of much needed drugs and vaccines^16^. The proposed oropharyngeal gonorrhoea CHIM will comprise of oropharyngeal inoculation via throat swab. Participants will be required to follow strict infection control procedures including abstinence from sexual activity including kissing and be monitored daily as an outpatient for several days. All participants will be treated with effective antimicrobial therapy. Participant inclusion criteria will be restricted to people assigned male at birth who do not have sex with people assigned female at birth. These strict CHIM participant inclusion criteria have been selected to mitigate the risk of transmission of gonorrhoea to people with a uterus (including due to inadvertent transmission due to protocol breaches that may occur in an outpatient CHIM), due to the potential long-term reproductive health consequences of gonorrhoea infection in this population.

When considering the development of a new STI-related CHIM, it is important to acknowledge the sociocultural and historical context in which the research is being proposed. Gonorrhoea CHIMs have not previously been performed in Australia and STIs remain highly stigmatized in Australia^17^, similar to most settings. The concept of experimental infection of humans with STIs draws associations with arguably some of the most well-known cases of ethical misconduct in the history of clinical research, including the United States (US)-led STI studies in Guatemala, involving non-consensual intentional infection of over 1,000 non-consenting Guatemalans with syphilis, gonorrhoea and chancroid (1946–1948)^18^; and the Tuskegee syphilis study, in which over 400 unconsenting rural African Americans individuals unknowingly infected with syphilis were denied effective treatment for decades (1932–1972)^19^. In line with modern clinical trial ethical principles as set out in the World Medical Association’s Declaration of Helsinki^20^, and the Australian National Statement on Ethical Conduct in Human Research^21^, any CHIM research must be conducted ethically and in careful collaboration with potential research participants^22^.

We report here results from a community consultation with potential research participants and gonorrhoea experts. In this consultation, we explored the perceptions of community members and expert stakeholders toward the development of an oropharyngeal gonorrhoea CHIM. Key barriers and enablers to participation and preferences for study design were also explored; these will be reported elsewhere.

## Methods

### Recruitment and sample

#### Community participants

Individuals were eligible if they met the self-identified eligibility criteria for participation in the proposed oropharyngeal gonorrhoea CHIM: (i) a cis-man aged 18–50 years with no chronic medical conditions; (ii) sexually active with other cis-gender men; and, (iii) lived in Victoria. The study was advertised using digital noticeboards and newsletters, physical posters and social media through affiliated sexual health, higher education and community organizations, with a call for eligible participants to provide their opinion about research that involved infecting people with gonorrhoea. Advertisements included a weblink and/or QR code that directed people to a REDCap screening survey, where participants could be screened for eligibility, provide contact information and indicate their preference for participation in an interview or focus group. Participants also provided demographic information to enable purposive sampling to be performed to ensure diversity amongst the sample. Those who expressed interest were contacted with further information about the study, and invited to participate in an interview or focus group as per their preference.

#### Expert participants

Individuals were eligible for participation if they self-identified as an expert with relevant knowledge in gonorrhoea infection, treatment or prevention; or if they self-identified as a community representative for priority populations at risk of gonorrhoea. Experts were identified through the investigators’ professional networks, with snowball sampling used to expand the sample size. In our sampling, we aimed to include participants from relevant clinical, industry (vaccine and pharmaceuticals) and community organizations. Experts were sent an initial email inviting them to participate in the study and were provided with further information about the study if they expressed an interest in participating.

### Data collection

**Fig. 1:**
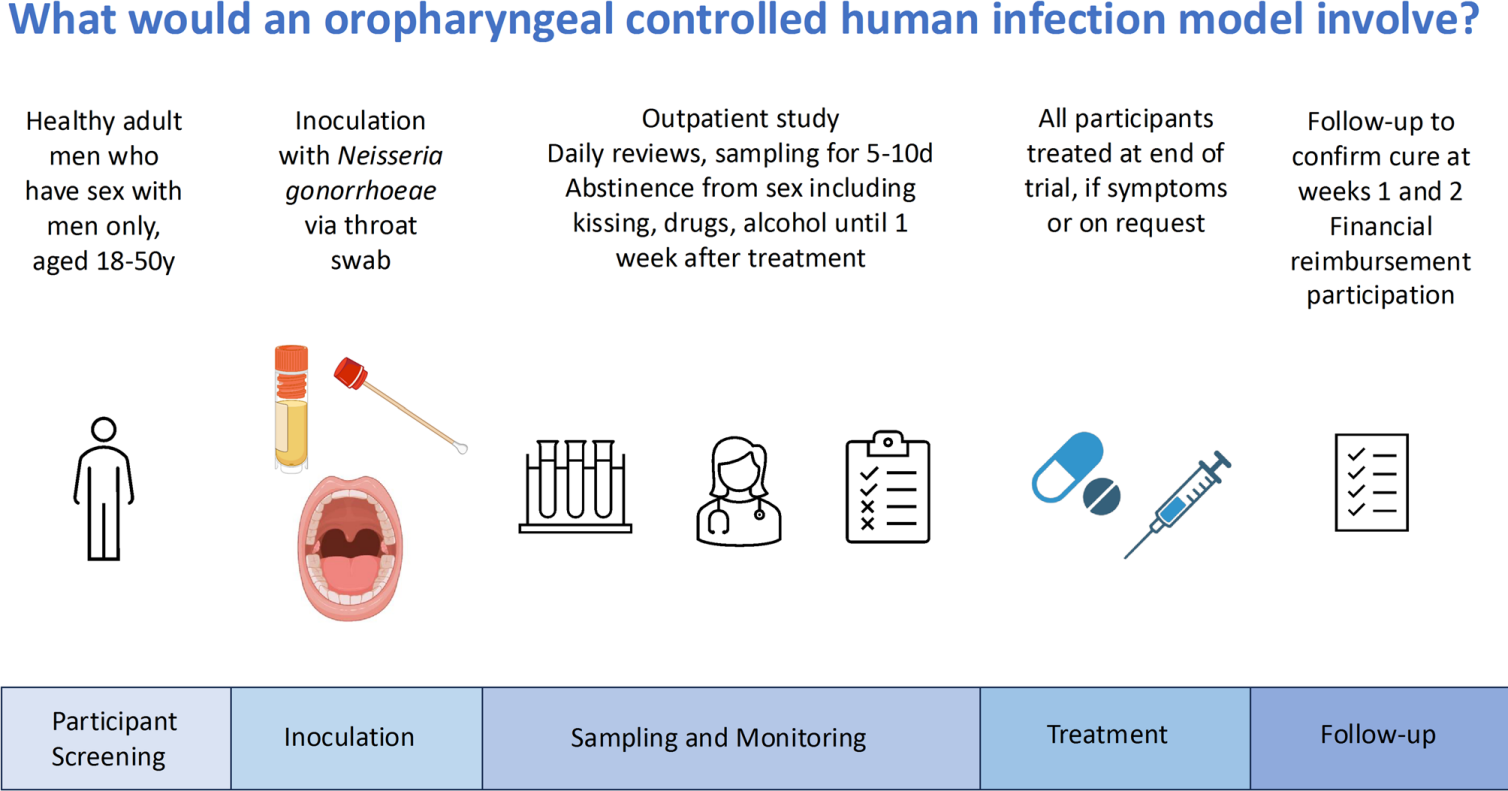
Schematic of proposed oropharyngeal gonorrhoea controlled human infection model study presented to participants.

The interviews and focus group were conducted via Zoom by JEM or EW using interview guides (Supplementary Appendix 1 and 2). After preliminary questions about participants’ understanding and views of gonorrhoea and other STIs, participants were provided with a brief description of clinical trials using CHIMs and an outline of the proposed oropharyngeal gonorrhoea CHIM study (Fig. 1; Supplementary Appendix 1 and 2). In brief, the proposed oropharyngeal gonorrhoea CHIM study comprises recruitment of healthy volunteers assigned male at birth who only have sex with other individuals assigned male at birth. These participants are then inoculated with *N. gonorrhoeae* via throat swab and attend daily outpatient monitoring reviews for five to ten days. After study completion, upon request, or if a participant develops symptomatic pharyngitis, they will be treated with curative antimicrobial therapy. All participants will be required to follow infection control procedures including sexual abstinence (including from kissing) throughout the five to ten days after inoculation and at least one week period after antibiotic treatment. Participants were then asked about their perceptions of the use of CHIMs as a clinical trial methodology, perceptions of gonorrhoea CHIMs and opinions around specific aspects of design and implementation of a gonorrhoea CHIM in the local setting. Data collection took place between July and November, 2024. Participant recruitment ceased when saturation was achieved. The duration of the interviews ranged between 25 and 73 min, while the focus group lasted 81 min. Participants received a $50 e-gift card as reimbursement for their time.

### Data analysis

Interviews and focus groups were professionally transcribed verbatim, de-identified, checked against the audio by EW and JEM, and uploaded into NVivo version 14 for data management and analysis. Data analysis followed the principles of thematic analysis^23^. EW and JEM read the transcripts, co-coded a representative sub-sample of transcripts and collaboratively developed a preliminary coding framework in consultation with LK. EW then coded the transcripts according to this framework and analysed the data relating to perceptions of community and expert stakeholders toward the development of an oropharyngeal gonorrhoea CHIM to develop sub-themes. Data from other codes will be reported elsewhere. Preliminary analysis was presented to LK and JEM, whose feedback was incorporated to finalise the themes and subthemes. The perceived acceptability (supportive, unsure or against) of the proposed oropharyngeal gonorrhoea CHIM and willingness to participate in the proposed study (willing, unsure, unwilling) was categorized for each participant, based on a holistic assessment of the overall interview/focus group contribution assessed independently by EW and JEM, with discrepancies resolved by consensus. The perceived acceptability, willingness to participate and demographic data were summarised numerically using descriptive statistics. This quantitative analysis approach was deemed necessary to assess the feasibility of the proposed oropharyngeal gonorrhoea CHIM.

### Ethics approval

Ethical approval was granted by the University of Melbourne Human Research Ethics Committee (2024-29587-54502-3), and approved by the Thorne Harbour Health Community Research Endorsement Panel (THH_24_018). All research was performed in accordance with the International Conference on Harmonization Good Clinical Practice Guidelines and in accordance with the Declaration of Helsinki. Informed consent was obtained from all participants.

## Results

### Participants

#### Community participants

161 people completed expressions of interest through the REDCap form during the study period. 122 individuals were deemed ineligible based on suspected provision of false information in order to receive the study reimbursement (e.g. use of celebrity names, provision of an invalid telephone number, identification of recruitment source that was no longer advertising and failing to respond to investigators’ attempts to confirm eligibility via telephone). Thirty-nine people were emailed with further information about the study and to confirm eligibility, two were deemed ineligible after further clarification and 15 declined after being given further information. In total, 22 community members participated in the study (Table 1). Data were obtained through 17 interviews and one focus group (comprising five participants) (will be referred to in text as C1 to C22).

#### Expert participants

Overall, 17 experts were emailed regarding participation, four were unable to be contacted and three declined. In total, 10 experts participated in the study, comprising professionals from various target expert groups including clinicians, industry representatives and community organization representatives (Table 1). All 10 individuals participated in a semi-structured interview (will be referred to in text as E1 to E10).

**Table 1:** Description of demographics and background knowledge and experience regarding gonorrhoea of participants.

Demographics	Expert participants (*n* = 10)		Community participants (*n* = 22)	
Age (years), median [IQR]	50 [40, 61]		31 [29, 36]	
Male, n (%)	7 (70%)		22 (100%)	
Employment, n (%)	Profession		Employment status	
	Sexual Health Clinician	2 (20%)	Unemployed	2 (9%)
	Infectious Diseases Clinician	3 (30%)	Student	5 (23%)
	Industry professional	3 (30%) (2 vaccine; 1 pharmaceutical)	Casual work	1 (5%)
	Community organization professional	2 (20%)	Part-time work	2 (9%)
			Full-time work	12 (55%)
Location, n (%)	Workplace location		Country of birth	
	Victoria	5 (50%)	Australia	9 (41%)
	Interstate within Australia	4 (40%)	Overseas	13 (59%)
	Outside of Australia	1 (10%)		
	Workplace category		Country of birth income level	
	Sexual Health Clinic	2 (20%)	High income	11 (50%)
	Hospital	3 (30%)	Upper middle income	9 (41%)
	Industry	3 (30%)	Lower middle income	2 (9%)
	Community representative organization	2 (20%)		
Highest level of educational attainment, n (%)	N/A		Certificate I-IV	1 (5%)
			Diploma/ Advanced diploma/Graduate diploma/certificate	3 (14%)
			Bachelor degree	8 (36%)
			Post-graduate degree	10 (45%)
Recruitment source, n (%)	N/A		Community organization	12 (55%)
			University	5 (23%)
			Sexual health clinic	4 (18%)
			Other	1 (5%)
Knowledge of gonorrhoea, n (%)	N/A		Excellent	3 (14%)
			Very good	6 (27%)
			Good	5 (23%)
			Limited	2 (9%)
			Minimal	6 (27%)
Disclosed history of gonorrhoea, n (%)	N/A		Yes	8 (36%)
			No	14 (64%)

N/A: not applicable (data not collected for expert participants).

### Summary of themes

Two themes related to acceptability of an oropharyngeal gonorrhoea CHIM were identified. The first was the potential perceived social and ethical issues associated with an oropharyngeal gonorrhoea CHIM, and the second the acceptability of an oropharyngeal gonorrhoea CHIM to the individual (Table 2). Narrative description of these themes and subthemes are presented below, with additional key quotes presented in the Supplementary Materials (Tables S1 and S2).

**Table 2:** Summary of themes and subthemes.

Theme	Subtheme	Key concepts
Perceived social and ethical issues	Related to inclusion/exclusion criteria	Inclusion of CALD individuals Inclusion of transgender and gender diverse individuals Inclusion of PLHIV Inclusion of people assigned female at birth and those that have sex with them Risk of stigma and discrimination related to participant inclusion/exclusion criteria
	Risk of stigma	Stigma related to infection with gonorrhoea Stigma related to participation in a CHIM study with gonorrhoea
	Complexities related to compensation and motivation for participation	Motivations for participation (personal including financial and future benefit; collective including community and scientific contribution) Ethical issues related to compensation for participation (undue inducement; appropriate reimbursement for contribution)
Acceptability of an oropharyngeal gonorrhoea CHIM to the individual	Overall acceptability	Factors associated with acceptability of proposed model Factors associated with willingness to participate in study Acceptability of oropharyngeal gonorrhoea compared to a gonococcal urethritis CHIM and CHIMs with other pathogens
	Perceived risks	Health risks Social risks (including stigma and impact on relationships) Public health (third party) risks
	Perceived benefits	Scientific benefit Advancement of novel interventions for gonorrhoea
	Influences on perception of the risks	Historical unethical STI human experiments Past personal experiences (STIs, clinical trials) Relationship with trusted institutions and ethics committees Importance of clinical governance and trial procedures (informed consent, support during trial, privacy and confidentiality)

CALD, culturally and linguistically diverse; CHIM, controlled human infection model; PLHIV, people living with human immunodeficiency virus; STI, sexually-transmitted infection.

1. Perceived social and ethical issues associated with an oropharyngeal gonorrhoea CHIM.
  1.1 Related to participant inclusion/exclusion criteria.
    Several expert and community participants highlighted that an oropharyngeal gonorrhoea CHIM should include a diverse range of participants to ensure that the findings of the study are generalizable to key populations affected by gonorrhoea, including people from cultural and linguistically diverse (CALD) backgrounds, transgender and gender diverse individuals, people living with HIV (PLHIV), and women. A minority of community participants were concerned that the inclusion of only MSM (who do not have sex with women) in the proposed oropharyngeal gonorrhoea CHIM may perpetuate stigma and discrimination targeted at MSM.
    Participants outlined the importance of equity of access for people to participate in clinical trials that influence their future healthcare, particularly in STI-related research. The inclusion of individuals frequently excluded from research, particularly culturally and linguistically diverse groups and transgender and gender diverse individuals was recommended.
    “*I would definitely take this opportunity to advocate for…including trans men in the study so that we can build a research evidence base to support that cohort.” (E3*,* community representative)*.
    Several experts identified PLHIV as a key population affected by gonorrhoea and encouraged the inclusion of PLHIV in the study, particularly individuals on antiretroviral therapy with a suppressed HIV viral load.
    *“If you exclude them…then your model’s not going to be actually able to be applicable to a decent chunk of people who are at risk of this infection*,* and require these results.”* (*E1*,* infectious diseases clinician)*.
    Community and expert participants highlighted that if inclusion of PLHIV was not possible, it would be important to communicate a clear scientific rationale for this exclusion criterion.
    It was acknowledged by experts that the exclusion of people with a uterus from participation may be a significant limitation to the generalizability of results to the key population impacted by long-term gonorrhoea morbidity. However, experts agreed that exclusion of people with a uterus was appropriate for the first human oropharyngeal gonorrhoea CHIM study.
    *“I think the place to start is with men only*,* no doubt*,* and to build up the experience and knowledge.” (*E5, industry representative).
    Most community members appreciated the reasons for exclusion of people with a uterus due to potential impacts on reproductive health
    *“You want to recruit…men because the side effects or the symptoms are not as serious for men as they are for women.” (C12)*.
    However, some community participants felt that people with a uterus could determine their risk tolerance depending on their personal preferences. As described by C9, “*what about women that have decided*,* ‘I don’t care about my uterus’?”* Based on the rationale that participants were required to be sexually abstinent during the trial, several community participants felt that heterosexual men should also be eligible to participate.
    Some community members identified ethical concerns with the exclusion of women and heterosexual men and associated this feature of study design with potential perpetuation of stigma and discrimination against MSM.
    “*My issue is segregating a part of demographic in terms of getting infection*.”*(C18)*.
    Concerns raised included: (i) a perceived lack of scientific justification for selecting from the MSM population and excluding other populations; (ii) the risk of the study perpetuating stigma and discrimination of a population that have faced historical and ongoing stigmatization; and, (iii) the potential wider social impacts of stigma and discrimination against MSM associated with advertisement of the study.
    “*You could argue the target population for years have faced stigma*,* discrimination*,* exclusion*,* marginalization.”* (E1, infectious diseases clinician).
    However, experts also identified protective factors associated with the generally high levels of health literacy, strong community and proactive healthcare engagement amongst the MSM population.
  1.2 Risk of stigma related to an oropharyngeal gonorrhoea CHIM.
    The risk of an oropharyngeal gonorrhoea CHIM increasing stigma was raised by several participants. This increased stigma could be related to the existing stigma associated with gonorrhoea itself or by the perceived stigma associated with a research participant agreeing to have their throat purposefully infected with gonorrhoea.
    Both experts and community participants identified that the initial response to a gonorrhoea CHIM may be characterized by rejection or even disgust, likely due to deeply embedded stigma associated with STIs.
    *“I think most people would think it’s like gross*,* and like*,* ‘Oh*,* my god*,* like people are getting their throats infected with gonorrhoea’.” (C3)*.
    This initial gut reaction was apparent even when participants were in support of a gonorrhoea CHIM study.
    “*I mean I think with an STI it feels a bit more icky somehow*,* doesn’t it*,* than something else? But it’s not really any different.” (E8*,* sexual health clinician)*.
    Participants perceived the broader community perception of STIs to be characterized by stigma and inherent blame of affected individuals, with some concerned about longer-term social risks.
    “*I think it may cause some reputation issues for yourself*,* right. So people think*,* well*,* this guy may have like multiple sex partners.” (C7)*.
    Several participants perceived a risk of stigma and discrimination related to participating in a study where they were voluntarily infected with an STI. Even individuals who felt free from stigma in their daily life described that they would feel uncomfortable sharing information about participation in an oropharyngeal gonorrhoea CHIM:
    *“I mean I’m pretty much an open book with a lot of people… Like I never feel any stigma. But I don’t know if it’s really something that I would like necessarily share with a lot of people that I participated.” (C15)*.
    Participants highlighted the importance of confidentiality and privacy for study participants to protect against this potential barrier to participation.
  1.3 Ethical issues related to compensation and motivations for participating in an oropharyngeal gonorrhoea CHIM.
    Personal and altruistic motivations for participation in the proposed oropharyngeal gonorrhoea CHIM were identified. Community participants felt that adequate financial compensation was a key factor that would influence their decision to participate. Identifying an appropriate level of financial compensation that would avoid undue inducement was described as an important ethical issue. Several participants felt that recruitment of participants with purely financial motivation could increase personal and third-party risks associated with the study. As such, identifying altruistic participants was highlighted as an important strategy to optimize the safety and ethical conduct of the study.
    Potential personal motivations for participation in the study included financial reimbursement and the potential for future health benefits arising from the study. Potential altruistic motivations for participation were related to contributing to the collective good within the MSM community and science at large. It was also highlighted that personal and collective motivations are not mutually exclusive.
    The majority of community participants indicated that financial reimbursement would impact their decision to participate. Participants generally agreed that adequate financial reimbursement was required to compensate for the time and other commitments involved in the study.
    “*If it’s not enough – and that*,* of course*,* varies depending on what people understand is enough or not enough – but if it’s not enough*,* I wouldn’t participate*.” *(C14)*.
    Several community member participants felt that because of the specific nature of this study there should be additional compensation afforded to participants for the social risks associated with the study, including the requirement for an extended period of sexual abstinence.
    *“Because it’s not just as simple as going and having your throat swabbed and check and check and check. There are then all those things to happen when you come home. Like no sex*,* no kissing*,* no glasses of wine*,* and whatever else could still come out of it.” (C16)*.
    Ethical issues related to compensation of individuals for participation in the study was a strong theme among experts and community participants. Several participants identified the difficulty of establishing an appropriate amount for financial compensation, that would strike an appropriate balance between reasonable reimbursement and undue inducement.
    *“I mean the other sort of ethical thing is around*,* if you’re paying people to do this sort of thing*,* whether it’s*,* for people of lower means*,* if it’s coercive. So I think I’d be setting that reimbursement rate at a rate that was enough to reflect their commitment in time*,* but not enough that you were putting undue coercive pressure on people who needed that money.” (E2*,* sexual health clinician)*.
    Experts identified that there may be a greater relative financial incentive for individuals of lower socioeconomic means to participate. Those who identified this ethical tension felt that it was important that the study recruit participants with altruistic motivations, noting that altruistic and financial motivators are not mutually exclusive. They suggested this could be achieved through a well-considered recruitment strategy.
    Several community and expert participants identified specific risks for protocol breaches that may arise from undue inducement and the recruitment of participants whose motivation for participation is primarily financial. These included: (i) concealing relevant medical history; (ii) concealing sexual orientation (i.e., sexual activity with women); and, (iii) being sexually active during the study. C3, who had previous experience of participating in clinical trials described how he had concealed important medical history:
    “*I used to have palpitations when I was a child. And they would be really annoying about letting me into the trials when they found that out. So I used to read the symptoms and make sure there was nothing in there about like heart. And then I would enrol without telling them that I had the palpitations*,* because I really needed the money at the time.” (C3)*.
    Due to the reliance on the participant to adhere to sexual abstinence to prevent gonorrhoea transmission, several expert and community participants remarked that recruitment of altruistic participants as an important risk mitigation measure.
2. Acceptability of an oropharyngeal gonorrhoea CHIM to the individual.
  2.1 Overall acceptability of the oropharyngeal gonorrhoea CHIM.
    Overall, 66% (21/32) of participants were supportive of the proposed oropharyngeal gonorrhoea CHIM, with 22% (7/32) unsure and 9% (3/32) against. Experts were more supportive of the oropharyngeal gonorrhoea CHIM, with 8/10 (80%) indicating that they perceived the study to be acceptable and 2/10 (20%) unsure. Uncertainties about the trial related to concerns regarding stigma, the tainted history of unethical STI-related challenge trials and difficulties associated with obtaining genuine informed consent for such a conceptually challenging study.
    Among community participants, 64% (14/22) were supportive, 23% (5/22) were unsure and 14% (3/22) were against the study. Those with better knowledge or personal experience of gonorrhoea were more frequently supportive (Fig. 2). Concerns identified amongst the group with less knowledge about gonorrhoea were the perceived health and social risks of the CHIM. Overall, people born in Australia, with a higher level of education and in current employment were more frequently supportive (Fig. 2).
    Among community participants, 45% (10/22) indicated a willingness to participate in the proposed study, 23% (5/22) were unsure and 32% (7/22) were unwilling (Fig. 2). Interestingly, the acceptability of the study did not translate directly into willingness to participate. People born in Australia and with an undergraduate level of educational attainment were more frequently willing to participate (Fig. 2). Of the 23% (5/22) of community participants that described a discordant attitude between the perceived acceptability of the study and their lack of willingness to participate, predominant barriers to participation related to risks to intimate partners, inadequate financial compensation and the impact of study requirements (sexual abstinence and inconvenience). By contrast, for the 27% (6/22) of community participants that described a concordance between their negative attitude towards the study and their lack of willingness to participate, barriers included perceived health risks, inadequate financial compensation and concerns regarding stigma and discrimination.
    **Fig. 2:**
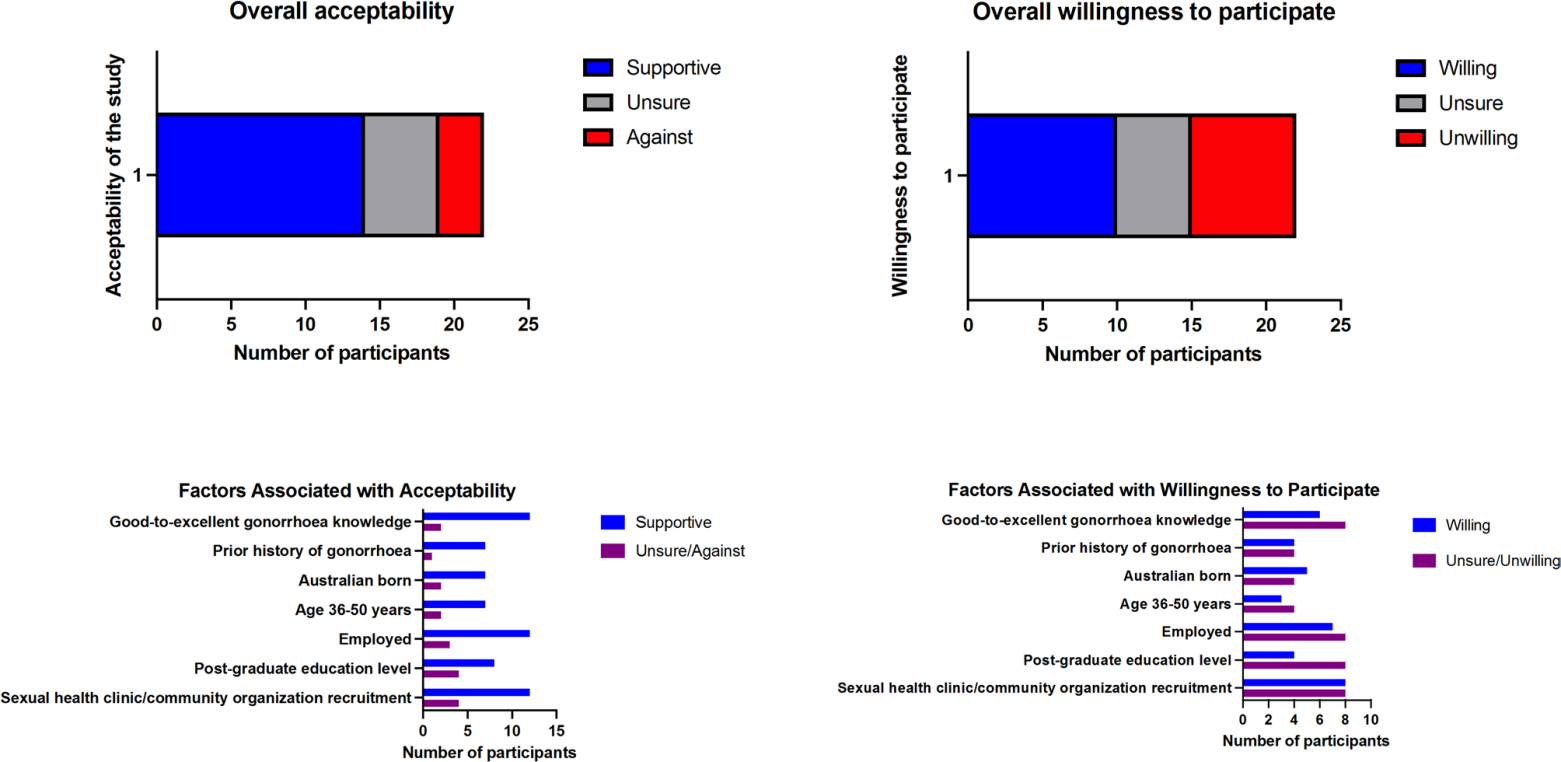
Community participants’ perceptions and willingness to participate in the proposed oropharyngeal gonorrhoea controlled human infection model study.
    Most participants agreed that an oropharyngeal model would be more acceptable than a urethral model. They cited anticipated discomfort of urethral instillation of gonorrhoea, vulnerability associated with both exposing their genitals to trial staff and health risks of gonorrhoea, including the increased risk of symptoms and other health risks as the rationale for this preference.
    “*You’re asking people to be vulnerable enough to expose their genitalia*,* have that genitalia messed around with in some particular way or form*,* and then the possibility of having symptoms.” (E3*,* community representative)*.
    As described by C3, the urethritis model was perceived as “*way less acceptable than the throat model*,* yes. I don’t think I would even do that. But I would consider doing the throat swab.*”
    Community participants had mixed views regarding the acceptability of an oropharyngeal gonorrhoea CHIM compared to a CHIM for a different infection (e.g. malaria, typhoid, influenza). Those who felt an oropharyngeal gonorrhoea CHIM was more acceptable felt that it was one of the lower risk CHIM studies. Conversely, although some community participants felt the risk of oropharyngeal gonorrhoea was lower than other infections such as malaria or typhoid, the perceived stigma associated with gonorrhoea meant some participants felt a gonorrhoea CHIM was less acceptable. Factors that would be weighed up when determining acceptability of a CHIM included familiarity with the infection, its curability, the route of infection, symptom likelihood and severity and its lifestyle impact. By contrast, for most experts, acceptability was similar across CHIMs:
    “*I don’t have thoughts around this bug versus another bug. They’re germs ultimately*,* to me as an infection doctor” (E1*,* infectious diseases clinician)*.
  2.2 Perceived risks.
    Most participants perceived the proposed oropharyngeal gonorrhoea CHIM to pose minimal health risks to potential participants. The potential social and public health risks of involvement in the study were identified as more prominent risks. In addition to stigma, these social and public health risks included impacts on relationships and transmission of gonorrhoea to other individuals.
    Experts agreed that there would be minimal health risks associated with an oropharyngeal gonorrhoea CHIM.
    *“I think the risks in that scenario*,* for the majority of people*,* are going to be virtually zero.” (*E2, sexual health clinician).
    While most community participants perceived the oropharyngeal gonorrhoea CHIM to be associated with minimal health risks, some were concerned about health risks, particularly related to unknown longer term health complications, persistent infection and AMR.
    *“So there could also be hidden costs*,* hidden consequences for the men having gonorrhoea. Like it could happen*,* anything*,* at some point in their lifetime*,* as well*,* and it isn’t still scientifically found at the moment.” (C4)*.
    Several community and expert participants were concerned regarding social risks associated with a gonorrhoea CHIM. The potential risk of transmission to close contacts, particularly sexual partners, was a prominent concern. Some participants felt that the sexual abstinence required for the study may have a detrimental impact on relationships. Compared to a requirement for abstinence from sexual activity involving the genitals, abstaining from kissing and potential transmission via contact with mouth or saliva were perceived to be more difficult by some participants.
    *“Like potentially abstaining from sex for a month is doable. But if someone has a partner and*,* say*,* you can’t kiss your partner for a month*,* that’s an issue.” (E9*,* community representative)*.
    Some community participants felt it would be important to discuss participation with their partner to ensure partners understood the rationale for behaviour change, but others felt that undertaking the study while in a sexual relationship would be too great a risk to the relationship.
    The public health risk due to third-party transmission was identified by expert and community participants as another key social risk. It was noted that protocol breaches related to sexual abstinence may occur through intentional deception or inadvertent protocol breach.
    “*I think it will either come from people not behaving in an ethical manner*,* which you can’t avoid*,* like people are going to be liars and careless. But*,* more often than not*,* I would suspect it’s going to come from people not paying attention to the instructions or being adequately given them.” (C15)*.
    Participants proposed that this risk required mitigation through education, clinical trial support and careful participant selection. Some community participants felt that this could be further mitigated by conducting an inpatient quarantine study. However, most perceived that community risks associated with an outpatient model would be minor and that trial procedures and regular STI screening would enable detection and treatment of inadvertently transmitted infections.
  2.3 Perceived benefits.
    The scientific benefits of the proposed model were acknowledged by most participants. It was highlighted that the development of new vaccines and therapeutics against gonorrhoea were of utmost importance and that translational studies to assess these should be the primary focus of this model.
    Strengths of the CHIM study identified by participants included the controlled nature of the infection, the opportunity to collect high quality data and the efficiency of the model for gathering these data.
    *“I’m a supporter of human challenge studies*,* as a clinical trial*,* to understand a lot of things…including pathogenesis and the immunological response to infections*,* right from the beginning of pathogen exposure or acquisition*,* up to the point of symptom development… in a very controlled manner.” (E10*,* infectious diseases clinician)*.
    Several participants noted that improved understanding of gonorrhoea arising from these studies could lead to improvements in treatment and prevention in the future.
    The potential for an oropharyngeal gonorrhoea CHIM to contribute to new interventions against gonorrhoea was identified by many participants. Several community participants qualified this by saying that the study had to be highly sensitive and performed within a robust ethical framework.
    *“I think this type of trial is useful if it’s regulated properly. I mean like it fast-tracks everything… So I feel yes*,* it’s good for the community*,* but I feel it has to be really treated with a lot of care.” (C10)*.
    Several experts felt that the model would be highly valuable and ethically justifiable due to the potential benefits it could have for vaccine and antimicrobial discovery. However, experts noted that field studies would still be required to test an intervention in a broader population.
  2.4 Influences on risk perception.
    Several factors were thought to play a role in participant perception of the risk of an oropharyngeal gonorrhoea CHIM. The association with historical unethical STI-related human experiments was a factor that increased the perceived risk of the study. Undertaking the study in partnership with trusted institutions and community organizations and according to modern ethical standards was perceived to improve confidence in the study.
    The main factor perceived to have a negative influence on risk perception was the association of an oropharyngeal gonorrhoea CHIM with historical unethical STI-related human experiments. The inherent association conjured by the proposal of an oropharyngeal gonorrhoea CHIM and historical atrocities was identified by both expert and community participants as a significant barrier to acceptability.
    *“So I mean it’s messed up but the first thing that came to my mind*,* like a very historical precedent*,* I think was like that scandal/crime against humanity that the government in the United States did*,* like the syphilis infection of the Tuskegee airmen*,* I think*,* without them knowing.” (C15)*.
    Participants felt that the instinctual wariness and suspicion invoked by this history could be mitigated by ensuring the oropharyngeal gonorrhoea CHIM is performed to the highest possible ethical standards and ensuring the study is designed with a participant-centred focus that is highly sensitive to this history.
    Multiple factors were perceived to have a positive influence on risk perception. Past personal experience of gonorrhoea and prior experience of involvement in research improved the perception of an oropharyngeal gonorrhoea CHIM. Endorsement or conduct of the study by trusted institutions was identified by several participants as having a key positive influence, including universities, healthcare institutions and community representative organizations. Affiliation with these institutions was described as providing “*a voice of trust and authority in our community to back it up” (C15)*. The pivotal gatekeeping role played by ethics committees was also highlighted.
    *“But I guess what it does also make me think about is the reliability of research standards in Australia and thinking*,* well*,* this would only be approved if it was all ethical to do by a lot of people that are a lot smarter than me.” (C19)*.
    Most participants felt that if an informed consent process was in place that enabled potential participants to volunteer for the study *“of their own free will” (E1*,* infectious diseases clinician)*, the oropharyngeal gonorrhoea CHIM would be *“perfectly reasonable and proportionate to the benefit to society” (E2*,* sexual health clinician).* However, other participants identified complexities in communicating the risk associated with this type of study to potential participants. It was felt that use of multi-modal communication in informed consent materials and recruitment of highly motivated, well-educated individuals with prior STI experience may improve the robustness of informed consent. Careful screening of participants, ready availability of medical support during the study, procedures for post-treatment follow-up and test of cure, and structures to ensure privacy and confidentiality for participants were also highlighted as important for reducing risk.

## Discussion

This study is the first to describe the perceptions of community and experts to gonorrhoea CHIMs in Australia, and one of two studies exploring the acceptability of STI-related CHIMs^24^. Due to the sensitivity and novelty of the proposed oropharyngeal gonorrhoea CHIM, this community consultation should guide future study designs of CHIMs for gonorrhoea^16^. As has been noted with other emerging clinical research areas such as HIV cure and novel HIV PrEP modalities^25,26^, this work demonstrates that key stakeholders and MSM community members are interested in novel research methodologies and want to have meaningful input into the design of research that impacts them.

Similar to a recent study investigating the acceptability of a gonorrhoea urethritis CHIM, stigma was a prominent theme^24^. As has been noted in research examining the negative impact of stigma on HIV and STI-testing uptake^17,27^, STI-related stigma^28^ identified in this study was identified as a potential barrier to participation in STI-related research. Stigma may pose a barrier to participation in an STI-related CHIMs, due to internalized self-stigma and fear of social harms related to public stigma associated with STIs^29^.

The inclusion of only individuals assigned male at birth who exclusively have sex with individuals assigned male at birth in the proposed oropharyngeal gonorrhoea CHIM, which is aimed at minimizing potential health risks related to infection in people with a uterus, was identified as a key social and ethical complexity. The history of stigma and discrimination experienced by MSM and potential for the proposed study to perpetuate sexuality-based stigma^30^ was highlighted as an important risk requiring mitigation. A highly sensitive and targeted recruitment strategy co-designed with community that clearly describes the scientific rationale for the study and restricted participant selection criteria could help to mitigate this risk.

Participants’ concern for other social consequences of participation, including risks of transmission to sexual partners and potential impacts on key relationships were other important findings. In qualitative research exploring the impact of persistent viral STIs, disclosure has found to be associated with fear and anxiety, altered sexual behaviour and can be a source of conflict in relationships^28,31^. Similar concerns regarding fear of relationship loss has been identified as an issue that influences decision-making in disclosure of gonorrhoea status^32^. Ensuring appropriate screening and treatment is available to partners during the study may help to mitigate concerns.

Appropriate financial compensation is an important ethical complexity in the design of any new CHIM. Investigators should aim to avoid undue inducement, e.g., payments that “prompt subjects to lie, deceive or conceal information that, if known, would disqualify them as participants”^33^. By contrast, due inducement is defined as payment that is “an established, reasonable fee-for-service schedule”, involving appropriate reimbursement for the time, procedures and/or inconvenience involved in participation^33^. Because of the reliance of participants to remain sexually abstinent during the study, participants with altruistic motivations rather than those with purely financial motivations were considered to be more trustworthy in mitigating the risk of gonorrhoea transmission to third-parties. Simultaneously, fair compensation relative to potential participants’ commitment to the study was seen as fundamental to successful recruitment.

Despite these complexities, the overall feasibility of the proposed oropharyngeal gonorrhoea CHIM has been reinforced by the finding that most participants were supportive of the study and almost half of the community participants indicated a willingness to participate. An oropharyngeal gonorrhoea CHIM was perceived to be more acceptable to participants than a urethritis CHIM, due to fears of discomfort, vulnerability and perceived health risks. Australian-born individuals, those with prior experience of gonorrhoea and those with links to sexual health clinics or community organizations were more frequently supportive and willing to participate. Recruitment strategies that enable access to this more highly motivated cohort may improve uptake.

This study has limitations that may affect the interpretation of these results. First, whilst the sample size exceeded recommendations for data saturation in qualitative research^34^, no new themes were emerging and repetition of earlier themes was observed, this study may not fully represent the views of all relevant stakeholders. This may have occurred due to potential selection bias, arising from the requirement for participants to volunteer to participate in the study. However, we achieved a diverse sample of participants with different ethnic backgrounds, educational levels and employment status in our recruitment. Second, community participation was restricted to the population that would be eligible for participation in the proposed study (MSM). Thus, the voices of other key populations impacted by gonorrhoea, including women, PLHIV, gender diverse individuals, sex workers and Indigenous Australians were not included in this sample, and would be important to guide potential broadening of inclusion criteria for future oropharyngeal gonorrhoea CHIM studies. Inclusion of experts and community representatives provided a wider perspective regarding the broader impacts of the study to these key populations. Finally, as participants self-selected for the study, those who participated may have had a greater interest in STI-related research than those that did not choose to participate. Despite these limitations, these data provide novel insights into key social, ethical and study design issues.

Embedding exploratory qualitative research in the overall proposed oropharyngeal gonorrhoea CHIM study program of work has enabled this community consultation to directly inform study design. This work demonstrates the fundamental role that community consultation plays in the design of contemporary research, particularly research associated with highly-stigmatized infections involving socially marginalized populations.

## Supplementary Information

Below is the link to the electronic supplementary material.

Supplementary Material 1

## Data Availability

The datasets used and/or analysed during the current study are available from the corresponding author on reasonable request.
