# Supplementary material for: Exploring community and expert perceptions of the acceptability of an oropharyngeal gonorrhoea controlled human infection model in Australia
Source: Sci Rep. 2025 Dec 2;16:1362. doi: 10.1038/s41598-025-30975-6 (PMC12796273; doi:10.1038/s41598-025-30975-6)
Supplement: Supplementary file 1 — Supplementary Material 1 [file 41598_2025_30975_MOESM1_ESM.pdf]

SUPPLEMENTARY MATERIALS

Table of Contents

Table S1. Perceived social and ethical issues associated with an oropharyngeal gonorrhoea controlled human infection model 2

Table S2. Acceptability of an oropharyngeal gonorrhoea controlled human infection model to the individual 10

Appendix 1. Interview and focus group schedule for community participants 19

Appendix 2 – Interview and focus group schedule for expert participants 23

Table S1. Perceived social and ethical issues associated with an oropharyngeal gonorrhoea controlled human infection model

**Subtheme**	**Key concepts**	**Expert participant quotes**	**Community participant quotes**
Related to inclusion/exclusion criteria	Inclusion of cultural and linguistically diverse individuals	*“Also cultural background perspective as well. And age diversity I think – because you’ve already mentioned 18 to 50 in there. So those other considerations around diversity of the participant… Yes, I mean you probably don’t want to – it would be a potential missed opportunity to recruit only Caucasian guys, for example.” (E3, community representative)*	-
		“And I suppose you want different ethnicities, ideally, don’t you, you know, just in case there’s anything different. So not just all white Australians or white Europeans. You probably won’t get any people who identify as Aboriginal or Torres Strait Islander, I would imagine, but I might be wrong. But maybe people from Asian countries. I don’t know whether there’s any proposed difference in – I don’t know of anything specifically in gonorrhoea and how it presents differently with different ethnicities.” (E8, sexual health clinician)	-
	Inclusion of trangender and gender diverse individuals	*“I think if you’re of populations at risk of gonorrhoea – throat gonorrhoea – then there would be transgender women in that population as well. And I look at it as a similar parallel to the people that they’ve done the Doxy PEP studies in, and the HIV Prep studies in. So pre-exposure prophylaxis. And that trans women is an important population as well. So I would certainly think about where that fits.” (E1, infectious diseases clinician)*	*“I still don’t know which one, if it’s a trans man or a trans woman, but anyway. But what about them? They’re like, “Well, I identify as a man. And now suddenly you’re saying that because I’m not male and only have sex with other men.” I think the community, in some ways, would be really happy to hear that something is now so applicable, healthcare related, to them” (C9, Australian born, interview)*
		“As a point, transgender men are frequently excluded or left out of research, which leaves transgender men in a – often in a precarious position in terms of there is no evidence to suggest that this community – and that’s because we haven’t studied it. So I would definitely take this opportunity to advocate for if it is possible to consider including trans men in the study so that we can build a research evidence base to support that cohort.” (E3, community representative)	

	Inclusion of people living with HIV	*"And I think it's actually really necessary because people living with HIV would make up a proportion of this population of gay and bisexual men who have sex with men. So I think if you exclude them, you are not going to be representative of this. And then your model's not going to be actually able to be applicable to a decent chunk of people who are at risk of this infection, and require these results. So I think that they definitely should be included." (E1, infectious diseases clinician)*	*"No issue with that either. There can be complications and different reasons for different results, depending on having multiple health conditions at one time. And treating and testing on a healthy population in the first instance makes sense." (C21, Australian born, focus group)*
		"Well, I think you'd want to look at CD4 counts or something like that. I wouldn't exclude them because they've got HIV. But I think you'd want to make sure that they were on adequate antiretroviral therapy and that their cell counts, their CD4 counts were OK. And, in fact, there's a good argument why you'd want to include them, because they are probably the population that you really want it to work in." (E6, industry professional)	*"Yes. I feel that my experience is generally not taking any other medication. So if someone is living with HIV, I mean that would seem to be quite risky to have to suspend that medication to participate in a clinical trial. That's what I would be concerned about if people living with HIV were included, I suppose."(C22, Australian born, focus group)*
		"It would really come down to whether we think that there's going to be further health impacts or the treatment would somehow be less effective. But similar to other gay men, HIV-positive gay men have a lot of sex and are getting treated for gonorrhoea at the moment. So I don't know if that would actually be different. I think if people with HIV were excluded, you'd need to be really clear and upfront on why. And so if it's a safety issue, just be really clear on that this is, "For these reasons, we don't want to take the risk of someone who's potentially immunocompromised getting the infection and having further negative impacts."(E9, community representative)	*"No. I was just wondering. And then the others kind of chimed in and added what I was going to – also, I agree with if the conditions are healthy, not with medical conditions or taking medications, that would make sense. But I assume if anything was then further down the years created out of this, like a vaccine, that would include everyone." (C19, overseas born, focus group)*
	Inclusion of women and heterosexual men	*"I mean we understand the safety issues, for example, of including women of reproductive age. But then that's a significant population where there (are) clinical consequences of infection. So that is a group where maybe this infection doesn't behave exactly the same. And it's actually a population that's very important to understand how gonorrhoea behaves." (E1, infectious diseases clinician)*	*"So for the other diseases, other infections, I think they are not as important and not – like they're important for all the people. But for this trial like it is more important – just going back to the gender divide, like they are more important for the women and the children, but not for the gay people I feel… Yes. But gonorrhoea could be more relevant if you could do the trial in women, not the gays. But for the malaria and other diseases you could do the trial on all the people. Like selection bias, based on their*

			gender, is a huge thing that we could consider."(C4, overseas born, interview)
		"I mean I understand why you'd want to develop a model only for women, right. I mean there are alternatives, right. I mean, for example, if you house the subjects in a phase 1 facility for two weeks, let's say, and they're not allowed to go home, you can do other things. Maybe you can actually include women. You'd have to be more careful for the reasons that you cited. But they'd be monitored all the time then for the couple of weeks that they are there, right. And if they have infection during that time, you treat them, right. So it reduces the overall risk, but it ends up being much more costly. And I know there are – as you know, I mean there are gender differences in infection and disease, right. So are we going to miss something by not having women in such a model?" (E5, industry representative)	*"Yes. But gonorrhoea could be more relevant if you could do the trial in women, not the gays. But for the malaria and other diseases you could do the trial on all the people. Like selection bias, based on their gender, is a huge thing that we could consider." (C9, Australian born, interview)*
		"No. I don't think so. I don't think you need to, really. I think you can do those experiments on men. I think we go through enough. We have a lot of reproductive morbidity to carry. We have all the pregnancy, all the contraception. I mean women do it all, quite honestly. I'm sure you appreciate this. But I don't think they need to be involved in that, no." (E8, sexual health clinician)	*"Well, I suppose, also, it's dependent on where the highest rates of infection are in the community. And I think... I know that there's a lot of very promiscuous gay men getting around. I don't know as to exactly whether there's the same infection rates in the straight women community. But the levels of transmission and the activity in a gay men's study I think is probably why it's – it wouldn't concern me for women not to be included, for that reason, also." (C16, Australian born, interview)*
	Risk of stigma and discrimination related to participant inclusion/exclusion criteria	*"I don't have thoughts around this bug versus another bug. They're germs ultimately, to me as an infection doctor. You could argue the target population for years have faced stigma, discrimination, exclusion, marginalisation. But also are extremely well engaged and often proactive populations, so men – gay and bisexual men who have sex with men." (E1, infectious diseases clinician)*	*"There'll be people that will say, 'Oh it's only homosexuals. It's not men' There'll be people that'll say, 'Why not women?'"(C9, Australian born, interview)*
		"Around the controlled infection there could be some hesitation. There would be questions, I think, from a lot of people. But given that it's a very small group that participates in this, I think it would be pretty easy to	*"However, my issue is segregating a part of demographic in terms of getting infection. I don't know how you will go through ethics to get this approved. Because if we're talking about genders, men and women – and if you're*

		reassure people that there's strong processes in place, it's a curable condition, no lasting impacts, all those sorts of things. So I think that there would be questions, but there wouldn't be outrage I don't think…Yes. So gay communities, yes."(E9, community representative)	*talking about penile, fine, we can't have women there. But if you're talking about oral, why can't women be included? It's not going to be a months-on-end trial. It's only going to be for a couple of weeks. And when you get consent, you can certainly just tell them appropriate birth control pills and so forth, contraception to avoid getting pregnant. That does happen with all the other trials." (C18, overseas born, focus group)*
			"Because when you said sexual health, it also came to the gay people. It always come to the gay people, right. I think it's because of the stigma of the HIV with gays." (C4, overseas born, interview)
Risk of stigma	Stigma related to infection with gonorrhoea	*"Most people would say gono is not a life-threatening, serious disease. Although it is, but it's perceived as a bit of a lifestyle disease. There might be some apathy around it, I don't know. Certainly there is in government circles. They don't take it very seriously and don't do anything about it, because they think it's like a disease you get when you go on a sex holiday and it's just a lifestyle disease. So you might have to deal with that issue" (E4, industry professional)*	*"I think a downside for me would – well, not a downside for me, personally. But a potential downside is the – so I have a marketing background, so I'm always thinking about the comms around this kind of thing. And I don't know, I can just see a headline in the Herald Sun about public money, or whatever it is, going into treating men who have sex – or giving men who have sex with men gonorrhoea to like find – you know -- and I just feel like that could spiral into some sex-negative story about the community and blah, blah, blah." (C17, Australian born, interview)*
		"But I would think the difficulty you're going to face is that gonorrhoea is not well known in the community. There will be a stigma about it."(E6, industry professional)	*"Like for example, if I'm a very healthy individual, I probably don't want to do it. Because in my mind, in my head, I would never get infected by those diseases. So that's my thoughts." (C1, overseas born, interview)*
		"So I wouldn't feel too optimistic about the general population. I mean a human challenge, yes, they'd be fine with, I think, if it was COVID or whatever. But not if it's an STI. That's how I feel. Because there's still a lot of homophobia. There's still a lot of blame and conservative approaches to STIs and things. We've got a long way to go still. We've come a long way, but we've still got a long way to go." (E8, sexual health clinician)	*"I think it would. I don't know if I can explain why. I mean I think getting yourself infected with an STI has a different connotation and sounds worse. I don't know if I can explain that well. But just it's different. And I don't know if you think – sorry, my brain's thinking and stuff. I think if it's like influenza, I think people would be really easily willing to do that because we've all had that at some point. If it's malaria, I think people would actually not be that interested because I think it sounds scary. So I think it*

			depends on how familiar people are and how scary it sounds, and I think gonorrhoea sounds scary." (C14, overseas born, interview)
			"Yes. I'm only talking from personal experience there. And I suppose the level of – I suppose you'd need, well, I think a level of maturity to consider putting yourself in such a situation. Because I know a lot of people don't react as calmly about STIs. And there's a lot of like panic around them and like unreasonable – well, what I think is unreasonable – thoughts around them. So I know just me personally with my comfortability with myself, that would have a lot to do with me deciding to be in it as well." (C13, Australian born)
			"There's something about getting swabbed with an STI in the throat that just makes me have like a gut reaction, even though it's irrational." (C3, Australian born, interview)
	Stigma related to participation in a CHIM study with gonorrhoea	*"If people spoke about their participation in the trial, they could experience stigma or discrimination. But I don't think that would be a really significant concern here. And, obviously, people are opting in, they're making this decision. And not everyone would tell everyone that, "Hey, I've been intentionally infected with gonorrhoea." So I don't think that's – it's something to be aware of, but I don't think it's a major issue."(E9, community representative)*	*"Because somebody, like if I'm talking to my friends or my family regarding this, it could be a bit of a – may be a type of issue. Or having to explain why or what it involves and entails, sometimes you may have to not say directly this and that. (C5, overseas born, interview)*
			"I think, yes, maybe a little bit. I mean definitely the initial gut reaction to it would be different if it was not an STI. And then if it was not – there's something about getting swabbed with an STI in the throat that just makes me have like a gut reaction, even though it's irrational. So maybe if it was like you're going to get infected with influenza via – actually, yes, I think it is something to do with the STI, and gonorrhoea specifically. Because if someone said that they were going to infect me via throat swab with influenza, for some reason it sounds so much less – I don't have nearly as much of a gut reaction to it. So I think there is

			something particularly about the word "gonorrhoea" (C3, Australian born)
			"So something that we've mentioned before is that I also weigh up the social like judgement if people know of it. Like of course I would try – like me, personally, I would not tell people. But just think about that, like what would people think if I had an STD? And like some people ask us, like ask people, other people, "Have you ever had an STD?" And if I partake in a trial and I say, "No," that would be a lie. And like that's something to consider, so like the social stigma." (C10, overseas born)
Complexities related to compensation and motivation for participation	Motivations for participation	*"Yes. In my mind that's kind of – and that may be also a bias of my own motivations. So it depends upon – different people can be motivated by different things. So yes, money can be one motivation or factor. The intrinsic motivation around, "Is it going to benefit me individually?" People may be motivated by whether it was going to benefit the people around them, their kind of cohort, their tribe effectively. So those are different motivational levers to pull, depending upon what you want to get. But certainly in a health promotion space, we often leverage both the individual motivation and the collective motivation as a two-pronged approach to be able to cover those kind of motivation factors around why we want some – why we are encouraging somebody to take action" (E3, community representative)*	*"I know there's like altruistic pros, that's it's going to help people and develop the vaccine and stuff. And maybe it's like particularly in the gay community or something. To be honest, that wouldn't appeal to me. Like if I was looking at it myself, the pros would just be like how easy is it, how much remuneration am I going to get." (C3, Australian born, interview)*
		"Yes. And I think the gay community and the MSM community are really used to – you know a lot of them are activists and they're really keen to help in all of these things. We see this in the clinic here. They really feel part of the problem and they want to help with the problem, and they sort of own it and run with it. I think after the days of HIV back in the '80s and things like that, that's been a tradition that continues, I think. So I think people could be recruited, yes." (E8, sexual health clinician)	*"Yes. In terms of my thoughts, like I think it would be a worthwhile thing. My thoughts about not knowing are just whether is that something I would consider. And I wouldn't do it – it would probably be a – like if it was financially worthwhile for me to do it, that would probably be my consideration. I wouldn't do it out of the goodness of my heart for like the community or for the benefit of people in the future. Like that, I suppose, would be my considerations." (C13, Australian born, interview)*
		"So I think if someone was adequately informed, it would be perfectly reasonable and proportionate to the benefit	*"Because for me, my intention for participation is just, because I am a man having sex with men, there are certain*

		to society, and potentially to the individuals involved, in terms of furthering the science for something that they're probably going to come from a community that's impacted by this problem." (E2, sexual health clinician)	*problems that play to the community at large. And then if I can participate and that participation helps provide insight to improving the overall health of men like me, I think that's a big win for me and the community. And there's people ahead of us who work – for example, like in HIV, as well, right, there was a lot of work that's been done by the people, which is why we have something like PrEP. It sounds simple, one tablet a day, but that has taken years of research and work, right. So I think we can all participate and provide whatever little we can into achieving the greater good. So once this is approved on an official scale, then I think there's no reason for me to not voluntarily involve myself, if I actually want to use it for the benefit of everybody around me." (C5, overseas born, interview)*
		"The idea of having a vaccine for gonno is something that most people would really welcome in the gay community. So I think from an altruism point of view – well, it's not pure altruism. But people participating in this because they see the benefit of it, both for their community and for themselves, I think you'd get a fair bit of interest in participating." (E9, community representative)	*"Yes. I do think, as well, the community broadly.... the queer community, that's certainly – well, I think about my group of people that are men who have sex with men, there is a community mindedness that people have. And so not everyone may want to take part in this for whatever reason, be it time or some other feeling about this kind of study, but people want to look out for each other. And whether you have had gonorrhoea or an STI, or not, I do think there is an understanding that that takes community, and community involvement even in this conversation, or community involvement in this study. I think that there is appetite for that." (C6, Australian born, interview)*
			"Well, personally, if I'm going to do that [the tria], I just want to do that in the name of science. I'm not going to charge anyone." (C9, Australian born, interview)
	Ethical complexities related to compensation for participation	*"But I think if you pay people enough, most people will do anything, really. It depends how much you're paying them." (E3, sexual health clinician)*	*"Yes, the time is compensated. And just like, you know – well, I like a glass of wine a couple of nights a week. So I would then have to go, "OK, well, I'm getting paid $1,500 not to have that glass of wine." So it adds up… Yes. Because it's not just as simple as going and having your throat swabbed and check and check and check. There are then all those things to happen when you come home. Like*

			no sex, no kissing, no glasses of wine, and whatever else could still come out of it." (C16, Australian born, interview)
		"However, if you're publicly promoting this study, then the likelihood is you're going to be more appealing to those people who are much more financially motivated. Which could be the very vulnerable people in the community who ultimately this is a money source for them and they need to do this for the money. So that is potentially ethically capitalising on their financial vulnerability. And that's the participants that you might get. So all of that kind of, 'How much is it? And how do people know about this' All of that kind of plays into this mix of, 'What is the right thing to do for community? And what is ethical, I guess?'"(E3, community representative)	*"But if you're going to offer financial compensation, and that is, in my view, probably what drives people the most to be part of this kind of test, you're going to be targeting people who are in more financially-marginalised situations because they want the money. And in Melbourne, for example, you're probably going to find a lot of men who were born overseas. And these are usually, from my knowledge, the ones who get less tested and the ones who are less connected within community-led health initiatives and all that. So there is this contextual thing that I think you have to think about, if that makes sense." (C14, overseas born, interview)*
		"And it's entirely appropriate that people should be remunerated in this kind of space. But then there's always that slightly tricky thing of is the remuneration – like what's the person's reasoning for doing it? Are they doing it because they want to contribute, they want to help, they want to find out? Or is it they're desperate for money and this is a way for them to get money? So it's not something that would stop this type of activity, research from happening. But it is just something that would need to be carefully considered in this type of model" (E9, community representative)	*"And then again, at the end of the day, if you have to give financial incentives, then is it really an informed, free consent? Because when you're giving financial incentives, then people will just say what you would want to hear." (C18, overseas born, focus group)*
			"I think if you pay too much you might be getting the wrong candidates. I think people that do this trial have to be interested in the reason behind the trial and the benefit that the trial results in. If people are just motivated by money and they're getting a good reimbursement, which is the way it's classified, and they're asked to abstain for 17 days, for example, well, there might be an incentive there to just go along because you're getting some nice money, and not actually comply with the trial. So I think by paying less you actually get people that are more committed. Controversial, but ..." (C21, Australian born, focus group)

<u>Table S2. Acceptability of an oropharyngeal gonorrhoea controlled human infection model to the individual</u>

Subtheme	Key concepts	Expert participant quotes	Community participant quotes
Overall acceptability	Acceptability of oropharyngeal gonorrhoea compared to gonorrhoea urethritis CHIM	*"I would guess, or I would wager that yes you would have a greater level of acceptance, or maybe frame it in the opposite of less likelihood of rejection if you're going with throat rather than penis. I think that there will be definitely a greater sensitivity and more questions asked from community in terms of, "What are you doing? Why are you doing this? And what is the consequence to me?" (E3, community representative)*	*"I think definitely the throat model. I mean the penile model is, number one, more invasive. Like not only would the administration be uncomfortable I reckon, not that I've tried, but like I just don't know what – yes. But also I think, to be honest, I don't know if the penile one or the throat one would have any long-term effects, but I feel the throat one would have less. I don't know why. I just feel like the throat one would have less effect than the genital one. Because when you say STD, like if it's in the sexual organs, then it's where it's meant to be, I guess." (C10, overseas born, interview)*
		"I think it would be much more difficult to recruit for a urethral challenge because I think there's a lot more discomfort associated with the urethral instillation. And I don't know the exact details, but I guess having worked in the hospital and inserting indwelling catheters and things, that's a little bit more involved. But they're certainly not comfortable and a lot of people find that quite distressing. So I think even just the idea of that would put a lot of people off. Whereas a pharyngeal swab is not something I think that people would automatically flinch against. Whereas a urethral instillation of gonorrhoea, people might just sort of – even just the thought of that, a lot of people might flinch and just not even consider reading on from there."(E10, infectious diseases clinician)	*"Look, I'd prefer the throat model because it's less invasive. Like if it were to be the urethral model, it would probably put me off a bit more participating, to be honest, than if it were the throat model, purely just because of like comfort I guess." (C12, Australian born, interview)*
			"I originally thought that it was a general sort of controlled infection, like a penile one. And so that wasn't overly palatable to myself. But knowing that it's a throat infection, less of a concern given that it's likely to be asymptomatic." (C21, Australian born, focus group)
	Acceptability of oropharyngeal gonorrhoea compared	*"It depends on how it's administered, I guess. Like I think malaria would be, I assume, intravenous, or you wouldn't be able to do that as a pharyngeal approach. So I think each one, I think a participant would judge that*	*"A good question. Those all sound a lot worse. Because I've had gonorrhoea before. So I mean like I don't think I would rank it in terms of malaria, typhoid or the flu. I've had the flu too. Gonorrhoea is easier than the flu. So if*

	to CHIMs with other pathogens	*each individually, I think, depending on how it's administered, the potential risks. But I wouldn't say necessarily that, say, malaria or typhoid – I don't think I would flinch on those particular infections or the mode of delivery of those particular pathogens. I think there's probably something more specific to urethral delivery of pathogens that is going to be less attractive to potential participants. Whereas an intravenous – like a blood test is something, again, that's pretty standard. So I think things that people are used to having done clinically, like blood tests, swabs, or if they have to ingest something, I think those things are pretty well understood and could be done" (E10, infectious diseases clinician)*	*you put all of those things on a list together, I'd pick gonorrhoea."(C15, overseas born, interview)*
		Well, I mean I guess malaria is much more likely to kill you. So do you mean in terms of the special stuff related to the STI, the stigma, all that sort of stuff? (E2, sexual health clinician)	*"If you're going to develop symptoms of typhoid, malaria or the flu, I'm not sure that is something that's overly palatable to myself."(C21, Australian born, focus group)*
			I don't know if I can explain why. I mean I think getting yourself infected with an STI has a different connotation and sounds worse. I don't know if I can explain that well. But just it's different. (C14, overseas born, interview)
			I think, yes, maybe a little bit. I mean definitely the initial gut reaction to it would be different if it was not an STI. And then if it was not – there's something about getting swabbed with an STI in the throat that just makes me have like a gut reaction, even though it's irrational. So maybe if it was like you're going to get infected with influenza via – actually, yes, I think it is something to do with the STI, and gonorrhoea specifically. Because if someone said that they were going to infect me via throat swab with influenza, for some reason it sounds so much less – I don't have nearly as much of a gut reaction to it. So I think there is something particularly about the word "gonorrhoea." (C3, Australian born, interview)
			For me if it's another disease, it depends on what we can and cannot do during the trial. As you mentioned, for this gonorrhoea you cannot kiss anyone. Which like for some people it's an important part of a relationship. So I believe

			it really depends on what you can and you cannot do during the whole trial." (C20, overseas born, focus group)
Perceived risks	Health risks	*"I think the downside is, obviously, the risks to the individual. But I mean with gonorrhoea there probably really isn't much risk to an individual. I mean I agree there is for women, so you wouldn't do it for them." (E8, sexual health clinician)*	*"But like potentially I'm not too sure if the treatment will affect the human health or have some like disadvantage to the human health or something. Because all the things are unknown, so I think this is one of my big concerns about the study." (C7, overseas born, interview)*
		"In terms of the risks, I do think that there's, obviously, no reproductive risks. Throat infection is broadly considered to be mainly asymptomatic. So I think the risks in that scenario, for the majority of people, are going to be virtually zero. I do think you would have to include in the patient information thing that risk of disseminated infection. So whilst that seems to be extremely low, it's not impossible. Like really weak data, but some of the guidelines do suggest it's more likely after throat. I don't know that's necessarily true, but it would be pretty hard to just ignore that." (E2, sexual health clinician)	*"Yes. I guess that would make it – because I'm young, probably if I saw that it was like self-limiting in most people, I would just assume that it would be in me. And then it would kind of make it – it makes it less serious, in my mind, like doing the trial." (C3, Australian born, interview)*
		"So I think the proposed protocol for the pharyngeal model seems acceptable to me and I think the community would be accepting of a protocol like that. And given the mode of inoculation is pretty – I would say is relatively harmless and it's just a swab. And then blood tests and follow-up testing all seems all pretty standard." (E10, infectious diseases clinician)	*"It feels really non-invasive. And I think the benefit of people having to go in every day after being given it is, again from a comms perspective, a way to show people – for people who are part of it to feel supported and safe, because there's that check-in every 24 hours of you going, "What are symptoms like? Are there symptoms?" et cetera." (C6, Australian born, interview)*
			"And I also think that, well, gonorrhoea is something that I have had before and there were no bad – but I have had the flu before and that was not something that had no effect." (C13, Australian born, interview)
	Social risks	*"Well, I think that goes back to the education that you'd be providing for participants. Do participants have a clear understanding of the reason we say not to kiss is not – we're not just saying don't kiss for any arbitrary reasons. It is specifically because kissing is a known possible vector of transmission of gonorrhoea. So therefore, if you are somebody that has a regular partner, and you want to participate in the study because you are*	*"I mean I'm fine. But because now I'm living with my boyfriend, so it depends on our understanding and our communication regarding to this period of time… Well, I mean in the relationship, I mean speaking from my own perspective, because we usually have it almost every day. And if I am supposed to not be having it like 10 or 12 or 14 days, I don't know what's my partner's thought about*

		motivated to do so, do you fully understand that you must abstain from kissing your partner during this time? And what does that mean? Is that a peck on the cheek? Is that a deep kiss, etc.?"(E3, community representative)	*that, and what is the effect after with like not having it for like 12 days." (C2, overseas born, interview)*
		"One point that you might need to consider is whether the person has a partner. So that's where the kissing issue comes in. Like potentially abstaining from sex for a month is doable. But if someone has a partner and, say, you can't kiss your partner for a month, that's an issue. So I don't know whether that would lead you down the direction of wanting single people. But I don't know, maybe people can make that decision for themselves." (E9, community representative)	*"Correct. Or talk about this with your sexual partners. Really consider the fact that you may not want – well, consider the fact that – strongly consider what it means to not have sex for two weeks. Is that compatible with your lifestyle? (C9, Australian born, interview)*
			"But I mean being in a relationship and having a partner, the other two more intimate aspects are part of daily life. And that's why I mentioned earlier on there would be a need to discuss that with them, as well, beforehand, because I think that could potentially impact the relationship environment." (C11, Australian born, interview)
			"Like if I had a partner at the time that would be a consideration, both for the abstinence and, I suppose, any risks that were associated from there kind of thing. I'd need them to be comfortable with it as well." (C13, Australian born, interview)
	Public health (third party) risks	*"And then I guess that goes into the same thing; that ties into recruiting the right people that can stick with the requirements of the trial. So it's an interesting thing in the sense that it's purely biological, right. So often in trials you want to get people who are from all different walks of life to make sure your sample reflects the reality of your clinic. Whereas in this scenario, I think you'd be focusing more on you just want people to stick to the study." (E2, sexual health clinician)*	*"Especially if it's highly transmissible, like relatively highly transmissible. I'm also worried that what if, in the course of the trial, I inadvertently give it to someone? Maybe not because of like kissing or anything, but maybe because of like saliva contact like from sharing food, drinking, something like that. Because the participants are sent home without any supervision, I think that's something that is possible. Just like a slip of the mind. Like just sharing food and then like, shoot, like I've been a part of this study and then – yes. So that's what I had in mind, actually, yes." (C10, overseas born, interview)*
		"The only other thing I can think of is, there is the possibility that people will not abstain for the full	*"In terms of the negative sides, which I think was the other part of the question, I mean I guess, again, a potential*

		amount of the study. Or even after they've started to take the treatment, they may jump in a little bit too early. So there is a possibility that this could lead to further infections in the community. But I think given there's only 26 people and I'm sure you'll give them plenty of support, that that risk can be managed." (E9, community representative)	*negative comes from people not doing the right thing during the study period and maybe potentially exposing others. Or engaging in activities, whether it's the alcohol or contact with others or whatever it might be, during that study period. Because it's, essentially, relying on them to do the right thing… Which is very different to, say, a controlled clinical trial where everyone's kept on premises and you've got more direct control over what they can and can't do and that sort of thing. So I guess the external environment, and then interacting with that, adds a degree of, I guess, risk." (C11, Australian born, interview)*
			"I think there is, of course, the issue that people might not commit to do it properly or not be careful not to have sex. Or even, as you said, potentially even kissing can transmit. So are people actually going to commit to that? But at the same time, I personally do not believe that it would have a massive impact on other people. Because as you said, this is very targeted for gay men, men who have sex with men. And I think that at least in places like Melbourne and in Sydney, I think men who have sex with men have really good awareness of sexual health. And people here do get treated. Like they get treated or get tested and treated very often, much more often than in other places that I've lived. So I think this would help mitigate any potential negative effects that someone not committing to not having sex or not going out and kissing other people would have. And I don't know if this, you know having someone who is not committing, would necessarily create a problem more than – or would increase my personal risk of getting gonorrhoea more than I already have." (C14, overseas born, interview)
Perceived benefits	Scientific benefit and pathogenesis	*"Sure. I mean on a personal level, I think it's wonderful to see that research in – dedicated research is moving in this direction for gonorrhoea because we already – it's already very clear that it's a problem. We're running out of solutions. So very encouraging to see that research is considering novel approaches to really tackle gonorrhoea and also focusing within a priority on the cohort of GBMSM in particular. So we are often lab rats.*	*"First of all, we can have a better understanding of gonorrhoea. Then, as I said, we can prevent this disease. I think, in general, we could improve the awareness, and also the health of the general public, by doing these trials." (C1, overseas born, interview)*

		But I think there is a community appreciation of a scientific interest in this space, and actually wanting to do good for our communities and the wider communities." (E3, community representative)	
		"The advantages are definite, in that you can control things. And so you can control infection and, therefore, better understand it and assess the efficacy of interventions and what is optimal because you're controlling the infection side of it. I think the benefits are massive, potential benefits."(E7, infectious diseases clinician)	*"I think it makes sense. Like I compare against what's the alternative, using animals. The ethical, moral quandary around consent and sentience and whatever, that's complicated. Humans can provide consent to this thing in a far more comprehensive way. And I think also.... particularly after things like COVID, and the understanding of wanting to develop things that are effective and can be proven in I guess a more holistic way, this kind of thing makes sense to me" (C6, Australian born, interview)*
		"So you get a lot of data from a small number of people having an infection. I think the quality of data you would get I would expect to be higher than to do an observational-type study. And it just sounds like it's a really efficient way to get that as well. So from a cost perspective, it would make a lot of sense." (E9, community representative)	*"Yes. I think the value is that it really gives you an opportunity to get a group of people together that actually consent and are knowingly infected with something, that you can sort of control and investigate the disease and effective treatment more closely. So I think it's like a perfect sort of environment where you can investigate that." (C12, Australian born, interview)*
	Advancement of novel interventions for gonorrhoea	*"Yes. I mean I think having a better way to prevent and treat gonorrhoea is certainly a high priority. And so, yes, I think it's a trial that would definitely meet the priority framework, or whatever you want to call it. And then particularly around that gonorrhoea AMR type stuff, if the controlled infection model can help with trialling different drugs, whether they're new drugs or repurposed drugs, then I think that would be absolutely justifiable." (E2, sexual health clinician)*	*"Yes. Then the population benefits would be good, because then you would be able to – it would presumably contribute to developing some sort of treatment or vaccine." (C3, Australian born, interview)*
		"I guess then the potential downside of that is that because it is so controlled, then if you are testing something, it may not necessarily be translatable to the general population. It could be us selecting for a very healthy group of people and providing treatments, or if we're doing treatments of vaccines in a small number of people. But I think as a very early-phase, exploratory nature of the study, it would be to determine whether	*"Yes, I do see some values. Because through these trials, you could have come up with some better strategies to cure them and also prevent that from happening. That's the value of it." (C1, overseas born, interview)*

		there's a signal for certain interventions that can then proceed to bigger trials, and then also determine the safety of them." (E10, infectious diseases clinician)	
Influences of perception of the risks	Historical unethical STI experiments	*"Yes. Exactly, yes. And you know, when I first brought the concept of controlled human infection model, the Tuskegee Syphilis Study was cited as a kind of reference of like, "We know the –" and that's human nature to kind of find the worst example of something. But that is a potential realistic backdrop that some community members may be evaluating this study against." (E3, community representative)*	*"Yes. I think initially it's quite confronting to know that we actively infect people with different diseases to then get better outcomes. I think it makes me really consider the trials that you hear about, I think it was maybe from the '60s I think, about the syphilis model in the US and how that – I think they were inmates maybe. Or some sort of patient was infected with syphilis and I think they ended up getting really sick. So a lot of these things come to mind."(C9, Australian born, interview)*
		"So, in general, I guess I feel concerned that we might be – so there's an inequity between a participant and a researcher that's often been a problem in medical trials in the past. So it's got a pretty ugly history. And I think my wariness mostly stems from that." (E7, infectious diseases clinician)	*"Honestly, I've actually never heard of like a trial where you are voluntarily inoculated – is that the word, inoculated or something – with a bacteria of this nature. So I mean it's messed up but the first thing that came to my mind, like a very historical precedent, I think was like that scandal/crime against humanity that the government in the United States did, like the syphilis infection of the Tuskegee airmen, I think, without them knowing. But I think that's just like a terrible historical precedent."(C15, overseas born)*
	Past personal experiences		*"I think so, especially knowing that it's throat. Because I think for all gay men who have already had throat gonorrhoea, it's always a surprise because you don't have any symptoms, you don't really know. So yes, I would probably – knowing that not having symptoms and that's kind of what is expected from you makes it easier." (C14, overseas born, interview)*
			"Because I've already taken part in the past with a flu trial where – yes. And even that – so maybe because I have a little bit of experience in a study already, I'm not too worried about potential side effects or getting symptoms." (C12, Australian born, interview)
	Relationship with trusted institutions and ethics committees		*"I think if the ethics committees approved... So once this is approved on an official scale, then I think there's no reason for me to not voluntarily involve myself, if I*

			actually want to use it for the benefit of everybody around me."(C5, overseas born, interview)
			"I think they're all universities or reputable organisations that are – it feels like it's being done for the community, not to create a new strain of drug to be used on the warfront or something. Like if there was a Defence Force logo on there somewhere, I'd be like, "What are we doing this for?" you know. But I feel generally supportive." (C6, Australian born, interview)
			"And so I think any type of – experimentation is a bad word, but you know, regarding our health and things that can actually hurt you is going to lead to I think naturally some suspicion and wariness. But there's plenty of respected doctors and organisations in our community that look after us and help us out who would be able to I think adequately share the availability of this study with people and be able to get the public support behind it." (C15, overseas born, interview)
			"Yes. Well, affiliations are important. And as I've indicated earlier, some of those brandings that were there on one of those slides, well, they're the types of affiliations that make me feel comfortable with the proposal." (C16, Australian born, interview)
	Importance of clinical governance and trial procedures	*"So I think an important thing in all clinical research is the informed consent process. And ultimately adults of their own free will to volunteer for these studies and be compensated for these studies – their time in these studies. So I don't have qualms from that point of view." (E1, infectious diseases clinician)*	*" I don't have any initial objections to the concept. I guess in terms of my initial reactions, I guess the important thing that comes to mind is the fully-informed consent of the people who are involved, and aware of potential outcomes in terms of the ways it could play out and the different end-points and whatever else, and I guess the follow-up and support." (C11, Australian born, interview)*
		"In terms of the ethics of it, I mean I think as long as someone is adequately informed, and limiting it to males, and even going further, in this case, to males without female partners, it does really remove the vast bulk of possible serious adverse events. So I think if someone was adequately informed, it would be perfectly reasonable and proportionate to the benefit to society, and potentially to the individuals involved, in terms of	*"Yes. I mean that was a gross oversimplification. I don't know what it would actually require. But I think the point I was making was maybe (a) put it in layman's terms for participants. But (b) just making it overwhelmingly clear, like this is what you must do to keep yourself and other people safe. And then also overwhelmingly clear what the risks are. But also why this is a good thing that they're*

		furthering the science for something that they're probably going to come from a community that's impacted by this problem." (E2, sexual health clinician)	*participating if you want to sell people on it" (C15, overseas born, interview)*
		"A bit hesitant about it. So, in general, I guess I feel concerned that we might be – so there's an inequity between a participant and a researcher that's often been a problem in medical trials in the past. So it's got a pretty ugly history. And I think my wariness mostly stems from that. Because although it's possible to inform people about the implications of infection and disease, I don't think it's ever possible to fully – the idea of fully, informed consent is, I think, quite difficult in this situation. And so that's something that concerns me. I'm not against them completely. I think they're a great opportunity to do a great many things. But the consent issue that's involved concerns me." (E7, infectious diseases clinician)	*"Yes. I think if you give proper counselling when you get consent, you really do tell them about the impact of it and whether or not they are OK to actually do that or not. And then again, at the end of the day, if you have to give financial incentives, then is it really an informed, free consent? Because when you're giving financial incentives, then people will just say what you would want to hear." (C18, overseas born, interview)*
		"I think support – I think follow-up support I think is kind of – we've touched on that already in terms of education and support, but also support in terms of like during the study protocol will people know who to contact, how to contact them etc.? You know, study – standard amongst study design." (C3, community representative)	*PP6 "What else would I consider? It doesn't worry me hugely, but I think the anonymisation part of it is a factor for people. There are lots of people who for various reasons, whether it be job or that they're not out, or whatever, would want to make sure that is really clear and that there's privacy around all of that. Again, I think in Australia that is perhaps an easier conversation than maybe other places, with established procedures and policies and whatever around that. [Pause] That's probably the main bits. And I guess the potential for adverse effects or for something to – an understanding of what support there is, or what happens if outside of hours something happens or that kind of thing. What's the support around that during that period, particularly because people are not in the clinic the entire time." (C6, Australian born, interview)*

Appendix 1. Interview and focus group schedule for community participants

This schedule is a guide for the semi structured interviews and focus groups conducted for the above project. The interviews and focus groups aim to cover the following questions, topics, and related issues, but note the wording and order may vary according to the facilitator, setting and participant. Information volunteered by the participants that doesn't directly pertain to a specific question should still be explored and noted at the end of the interview.

Introduction

Thank you for volunteering your time today to complete this interview and participate in our study. The interview will focus on your experiences and attitudes toward the sexually-transmitted infection, gonorrhoea, and your attitude towards implementation of a research model of controlled human infection with gonorrhoea in Victoria. We will discuss in further detail what a controlled human infection with gonorrhoea is later in this discussion.

At this point I would just like to reassure you that there are no right or wrong answers and if there is a question you are not comfortable answering you can skip it. Your participation in this study is voluntary and as such you may stop the interview at any time or withdraw from the study up until data analysis has occurred.

This interview will be recorded and transcribed. However, please feel free to give your honest opinions as you can be assured your name and contact details will be stored separately to the recordings. In addition, the transcript of the interview will be de-identified by removing any personal information from the transcript and any quotes we use in publications or presentations.

Any questions or concerns before we start the interview?

The recorder is now on

I will start with some preliminary questions about your understanding of gonorrhoea infection and then provide some further information about controlled human infection models in general and those proposed for gonorrhoea specifically.

Interview Questions

Part 1 – Understanding of gonorrhoea infection

1. Can you tell me a bit about what you understand about gonorrhoea infection
2. Is gonorrhoea something that concerns you? More or less so than other STIs?
3. Can you tell me how you manage your sexual health? (interview only)
4. What have you heard about effective strategies to prevent the transmission of gonorrhoea?

Background

I will now provide you with some background information on the further topics we will be discussing. If you have any questions or want to bring anything up at any point, please feel free to do so.

A controlled human infection model involves deliberate exposure of participants to an infectious agent in order to improve knowledge of a disease or enable testing of new treatment and prevention strategies. These models enable testing of a new strategy, such as a new vaccine, to determine whether it can prevent disease. By using a controlled human infection model, a small number of individuals are deliberately exposed to an infection and the research team know that there is a high chance that the individual would have developed an infection if they didn't receive the vaccine. This approach enables exposure of a much smaller number of people to the new

vaccine. The other approach to testing a new strategy like a vaccine involves field studies which require the involvement of thousands of people, as these people are at a much smaller risk of developing the infection in their daily lives. Controlled human infection models have helped to fast-track vaccines for typhoid, cholera, malaria and RSV and new drugs for RSV and malaria. These studies have been reviewed and approved by independent human research ethics committees around the world and serious adverse events in trial participants are rare.

Gonorrhoea is a sexually transmitted infection caused by the bacterium, *Neisseria gonorrhoeae.* It is a major global public health problem infecting 87 million people per year. Infection with gonorrhoea is becoming more common in Australia and other high-income countries around the world. In Australia the number of gonorrhoea infections has increased by approximately 90% in the past 10 years. Gonorrhoea can cause a wide variety of disease, ranging from infection without symptoms at the throat, mouth and anus, to infection of the genitals that can cause pain and discharge. It rarely causes a disease that affects the joints, skin, heart or lining of the brain and spinal cord. If not diagnosed and treated early, gonorrhoea can lead to severe outcomes, which have a greater impact on women and children including infertility. Infection also increases the risk of developing HIV. Resistance to antibiotics is another important global challenge in gonorrhoea infections. The *Neisseria gonorrhoeae* bacterium is very good at developing resistance to antibiotics and has developed resistance to all available antibiotics used to treat it, including the current first-line drugs used around the world.

Researchers at the University of Melbourne have proposed the development of a gonorrhoea controlled human infection model of the throat to improve prevention and treatment gonorrhoea. A throat gonorrhoea controlled human infection model would help with testing of new drugs, as infection of the throat is the most difficult site to cure. There is also increasing interest in the development of a gonorrhoea vaccine, however modelling studies suggest that a gonorrhoea vaccine would have limited impact on gonorrhoea levels in the community unless it was effective at the throat. There is increasing evidence that suggests that throat gonorrhoea is important in increasing transmission of gonorrhoea from person to person, so it is important that a vaccine is effective against throat gonorrhoea to reduce circulation of gonorrhoea in the community. As throat gonorrhoea is usually readily treatable and results in no symptoms or long-term health effects if treated early, a throat gonorrhoea controlled human infection model performed according to rigorous ethical standards may offer an opportunity for research that improves the future of gonorrhoea treatment and prevention with minimal risks to study participants.

This would be the first time that a controlled human infection model of gonorrhoea would be undertaken at the throat, however a male controlled human infection model of gonorrhoea of the penis has been performed in the United States since the 1980s with several hundred participants taking part and no significant adverse events. This model involves enrolment of healthy adult male participants aged 18-35 years old. Participation involves instillation of *Neisseria gonorrhoeae* via a small catheter placed 5cm into the penis and samples of blood, urine, and penile swabs being collected over a 5-10 day period. Participants attend the trial centre daily but return home to sleep and undertake their usual activities around the clinical trial commitments. They need to abstain from sexual activity, drugs and alcohol for the duration of the study and a 1 week period after antibiotic treatment. At the end of the trial, upon request or at the development of penile discharge, participants are given antibiotics to treat gonorrhoea infection. It is expected that the majority of people who become infected would experience symptoms of urethritis such as penile pain/discharge or burning with urination. All participants will undergo follow-up visits at 1 and 2 weeks after treatment for further sampling to confirm cure of the infection. Participants are financially reimbursed for their time for involvement in this study.

A controlled human infection model of throat gonorrhoea study would involve enrolment of healthy 18-50 year old male participants that identify as men who have sex with men and do not have sex with women. Participation would include having the *Neisseria gonorrhoeae* bacteria swabbed onto the back of the throat and samples of blood, saliva, throat swabs, urine and rectal swabs over a 5-10 day period. Participants would spend the first day in the clinical trials centre and attend the clinical trials centre each morning for 1-2 hours. They would return home to sleep and undertake their usual activities around the clinical trial commitments. Participants would need to abstain from sexual activity including kissing, as well as drugs and alcohol for the duration of the study and a 1 week period after antibiotic treatment. At the end of the trial, on request, or in the event of signs or symptoms of pharyngitis (symptomatic throat infection), participants would be given antibiotics to treat gonorrhoea infection. It is expected that the majority of people with throat infection will remain symptom free. All participants will undergo follow-up visits at 1 and 2 weeks after treatment for further

sampling to confirm cure of the infection. Participants would be financially reimbursed for their time for involvement in this study.

Importantly, neither the penile nor throat controlled human infection models will be undertaken in women. Cis-gender women have been purposely excluded from participating in this model due to the individual adverse reproductive health sequelae associated with gonorrhoea infection in women. In addition, recruitment procedures are restricted to reduce the risk of transmission gonorrhoea from participants to others, and particularly to women, during the trial, including recruitment of men who have sex with men as participants and abstinence from sexual activity including kissing of participants.

Today's interview/focus group will look into your attitude towards implementation of a research study involving a controlled human infection model of throat gonorrhoea in Victoria. I will be asking you questions about your opinions on a broad range of topics including

- Controlled human infection model studies
- Proposed implementation of a gonorrhoea controlled human infection model in Victoria
- Key questions in gonorrhoea controlled human infection model design

Please note that the throat gonorrhoea controlled human infection model discussed today has not yet been approved by a human research ethics committee and the design of this study is in progress. However, many studies using this controlled human infection model design have been approved by human research ethics committees and undertaken in various sites around the world with a number of different infections, including malaria, typhoid and COVID-19. Your responses will provide researchers with information about the opinions of potential participants regarding this proposed model with the aim that this will enable the model to be designed in an appropriate manner that is acceptable to the community. We ask that you give us your honest opinions as the information you provide us with today will be very valuable in highlighting the perspectives of potential participants in this study.

Do you have any questions? Y/N

End presentation

Part 2 – Understanding and views of controlled human infection model studies and their use in research

As discussed earlier, controlled human infection models have been used as a research tool with the potential to be implemented to improve understanding of disease and accelerate the development of new antibiotics and vaccines.

1. Can you tell me your initial reaction to this?
2. Is this something new, or have you heard about it before? (If so, where from? Who have you discussed it with?)
3. Do you see any value in this type of study?
4. Do you see any downsides to this sort of study?

Part 3 – Perceptions of controlled human infection model studies for gonorrhoea

As discussed earlier, researchers at the University of Melbourne are proposing a controlled human infection model of throat gonorrhoea.

1. Can you tell me your initial reaction to the idea of this study of throat gonorrhoea being undertaken in Victoria?

2. Does it bring up any concerns for you?

3. What do you think the public health benefits might be?

4. What do you think the community reaction might be?

5. Can you imagine what you would be most concerned about if you were asked to participate in this research?

6. How would you decide whether to participate? What would you weigh up?

7. Would you get advice from anyone before taking part?

8. Would the decision be different if you were asked to be part of a controlled human infection model study for another infection, like malaria, typhoid or influenza?

Part 4 – Opinions around the design of a gonorrhoea controlled human infection model

I am keen to get your views on specific aspects of the study design.

1. If you were asked to take part in a gonorrhoea controlled human infection model, would you have a preference for either the throat or penis model?

Requirements of participation

As discussed, participants of an throat gonorrhoea controlled human infection model would need to abstain from kissing, sex and drugs and alcohol for the period of infection and 7 days after treatment of the infection as per routine STI guidelines. This is to ensure that people who are not participating in the trial (such as partners) are not exposed to gonorrhoea infection.

2. How would you feel about not having sex/kissing/taking drugs and alcohol for 14 days? What about for 12 days? Or 17 days?
3. Would this be easy to adhere to?

4. Would it put you off participating?

Treatment

The standard treatment for gonorrhoea is an antibiotic injection in the gluteal muscle and an antibiotic tablet. There is also a different antibiotic tablet that could be used for treatment in this model, rather than the injection plus antibiotic treatment if tests indicate that the infection is susceptible.

5. Would the type of treatment being offered affect your participation (e.g. tablet versus injection?)

Payment

6. We propose to offer approximately $1500 for participation in this study. Do you think this would be an acceptable reimbursement for participation in this study?

7. Would the amount of money being offered affect your decision to participate?

Information

8. What information do you think is important to include in information and consent materials to help potential participants to understand the risks and benefits of the study?

Final Comments

This brings us to the end of the set of questions for the interview.

1. Is there anything we have not covered today that you would like to comment on?

End of Interview

That's all the questions we have for you today. I'm going to turn the audio recorder off to end the formal interview/focus group process. Thank you very much for volunteering your time today. The answers you provided will give us valuable insight into the acceptability of a controlled human infection model for gonorrhoea and will be important in guiding future research in this area.

Appendix 2 – Interview and focus group schedule for expert participants

This schedule is a guide for the semi structured interviews and focus groups conducted for the above project. The interviews and focus groups aim to cover the following questions, topics, and related issues, but note the wording and order may vary according to the facilitator, setting and participant. Information volunteered by the participants that doesn't directly pertain to a specific question should still be explored and noted at the end of the interview.

Introduction

Thank you for volunteering your time today to complete this interview and participate in our study. The interview will focus on your experiences and attitudes toward the sexually-transmitted infection, gonorrhoea, and your attitude towards implementation of a research model of controlled human infection with gonorrhoea in Victoria. We will discuss in further detail what a controlled human infection with gonorrhoea is later in this discussion.

At this point I would just like to reassure you that there are no right or wrong answers and if there is a question you are not comfortable answering you can skip it. Your participation in this study is voluntary and as such you may stop the interview at any time or withdraw from the study up until data analysis has occurred.

This interview will be recorded and transcribed. However, please feel free to give your honest opinions as you can be assured your name and contact details will be stored separately to the recordings. In addition, the transcript of the interview will be de-identified by removing any personal information from the transcript and any quotes we use in publications or presentations. However, given the strong network of researchers and stakeholders in this area, please be advised that you may be identifiable to others working in this area on the basis of views you express.

Any questions or concerns before we start the interview?

The recorder is now on

I will start with some preliminary questions about your understanding of gonorrhoea infection and then provide some further information about controlled human infection models in general and those proposed for gonorrhoea specifically.

Interview Questions

Part 1 – Understanding and views of gonorrhoea infection

1. Can you tell me a bit about what you understand about gonorrhoea infection?
2. Is gonorrhoea something that concerns you? More or less so than other STIs?
3. What is your opinion regarding effective strategies to treat gonorrhoea and prevent its transmission?

Background

I will now provide you with some background information on the further topics we will be discussing. If you have any questions or want to bring anything up at any point, please feel free to do so.

Note: this information will be modified according to the expert/community representatives' background knowledge and level of expertise. The interviewer will be confident to make these adjustments throughout the interview as it proceeds.

A controlled human infection model involves deliberate exposure of participants to an infectious agent in order to improve knowledge of a disease or enable testing of new treatment and prevention interventions. These

models enable testing of a new intervention such as a new vaccine, to determine whether it can prevent disease. By using a controlled human infection model, a small number of individuals are deliberately exposed to an infection and the research team know that there is a high chance that the individual would have developed an infection if they didn't receive the vaccine. This approach enables exposure of a much smaller number of participants to the new intervention. The other approach to testing an intervention like a vaccine involves field studies which requires the involvement of thousands of people, as these people are at a much smaller risk of developing the infection in their daily lives. Controlled human infection models have helped to fast-track vaccines for typhoid, cholera, malaria and RSV and drugs for RSV and malaria. These studies have been reviewed and approved by independent human research ethics committees around the world and serious adverse events in trial participants are rare.

Gonorrhoea is a sexually transmitted infection caused by the bacterium, *Neisseria gonorrhoeae.* It is a major global public health problem infecting 87 million people per year. Infection with gonorrhoea is becoming more common in Australia and other high-income countries around the world. In Australia the number of gonorrhoea infections has increased by approximately 90% in the past 10 years. Gonorrhoea can cause a wide variety of disease, ranging from infection without symptoms at the throat, mouth and anus, to infection of the genitals that can cause pain and discharge. It rarely causes a disease that affects the joints, skin, heart or lining of the brain and spinal cord. If not diagnosed and treated early, gonorrhoea can lead to severe outcomes, which have a greater impact on women and children, including infertillity. Infection also increases the risk of developing HIV. Resistance to antibiotics is another important global challenge in gonorrhoea infections. *The Neisseria gonorrhoeae* bacterium is very good at developing resistance to antibiotics and has developed resistance to all available antibiotics used to treat it, including the current first-line drugs used around the world.

Researchers at the University of Melbourne have proposed the development of a gonorrhoea controlled human infection model of the throat to improve prevention and treatment of gonorrhoea. A throat gonorrhoea controlled human infection model would help with testing of new drugs, as infection of the throat is the most difficult site to cure. There is also increasing interest in the development of a gonorrhoea vaccine, however modelling studies suggest that a gonorrhoea vaccine would have limited impact on gonorrhoea levels in the community unless it was effective at the throat. There is increasing evidence that suggests that throat gonorrhoea is important in increasing transmission of gonorrhoea from person to person, so it is important that a vaccine is effective against throat gonorrhoea to reduce circulation of gonorrhoea in the community. As throat gonorrhoea is usually readily treatable and results in no symptoms or long-term health effects if treated early, an throat gonorrhoea controlled human infection model performed according to rigorous ethical standards may offer an opportunity for research that improves the future of gonorrhoea treatment and prevention with minimal risks to study participants.

This would be the first time that a controlled human infection model of gonorrhoea would be undertaken at the throat, however a male controlled human infection model of gonorrhoea of the penis has been performed in the United States since the 1980s with several hundred participants taking part and no significant adverse events. This model involves enrolment of healthy adult male participants aged 18-35 years old. Participation involves instillation of *Neisseria gonorrhoeae* via a small catheter placed 5cm into the penis and samples of blood, urine and penile swabs being collected over a 5-10 day period. Participants attend the trial centre daily but return home to sleep and undertake their usual activities around the clinical trial commitments. They need to abstain from sexual activity, drugs and alcohol for the duration of the study and a 1 week period after antibiotic treatment. At the end of the trial, upon request or at the development of penile discharge, participants are given antibiotics to treat gonorrhoea infection. It is expected that the majority of people who become infected would experience symptoms of urethritis such as penile pain/discharge or burning with urination. All participants will undergo follow-up visits at 1 and 2 weeks after treatment for further sampling to confirm cure of the infection. Participants are financially reimbursed for their time for involvement in this study.

A controlled human infection model of throat gonorrhoea study would involve enrolment of healthy 18-50 year old male participants that identify as men who have sex with men and do not have sex with women. Participation would include having the *Neisseria gonorrhoeae* bacteria swabbed onto the back of the throat and samples of blood, saliva, throat swabs, urine, rectal swabs over a 5-10 day period. Participants would spend the first day in the clinical trials centre and attend the clinical trials centre each morning for 1-2 hours. They would return home to sleep and undertake their usual activities around the clinical trial commitments. Participants would need to abstain from sexual activity including kissing, as well as drugs and alcohol for the duration of the study and a 1 week period after antibiotic treatment. At the end of the trial, on request, or in the event of signs or

symptoms of pharyngitis (symptomatic throat infection), participants would be given antibiotics to treat gonorrhoea infection. It is expected that the majority of people with throat infection will remain symptom free. All participants will undergo follow-up visits at 1 and 2 weeks after treatment for further sampling to confirm cure of the infection. Participants would be financially reimbursed for their time for involvement in this study.

Importantly, neither the penile nor throat controlled human infection models will be undertaken in women. Cis-gender women have been purposely excluded from participating in this model due to the individual adverse reproductive health sequelae associated with gonorrhoea infection in women. In addition, recruitment procedures are restricted to reduce the risk of transmission gonorrhoea from participants to others, and particularly to women, during the trial, including recruitment of men who have sex with men as participants abstinence from sexual activity including kissing.

Today's interview/focus group will look into your attitude towards implementation of a research model of controlled human infection with gonorrhoea in Victoria. I will be asking you questions about your opinions on a broad range of topics including

- Controlled human infection model studies
- Proposed implementation of a gonorrhoea controlled human infection model in Victoria
- Key questions in gonorrhoea controlled human infection model design

Please note that the throat gonorrhoea controlled human infection model discussed today has not yet been approved by a human research ethics committee and the design of this particular study is in progress. However, many studies using this controlled human infection model design have been approved by human research ethics committees and undertaken in various sites around the world with a number of different infections, including malaria, typhoid and COVID-19. Your responses will provide researchers with information about the opinions regarding this proposed model with the aim that this will enable the model to be designed in an appropriate manner that is acceptable to the community. We ask that you give us your honest opinions as the information you provide us with today will be very valuable in highlighting your perspectives on this study.

Do you have any questions? Y/N

End presentation

Part 2 – Understanding and views of controlled human infection model studies and their use in research

As discussed earlier, controlled human infection models have been used as a research tool with the potential to be implemented to improve understanding of disease and accelerate the development of new antibiotics and vaccines.

1. Can you tell me your initial reaction to this?
2. Is this something new, or have you heard about it before? (If so, where from? Who have you discussed it with?)
3. Do you see any value in this type of study?
4. Do you see any downsides to this sort of study?

Part 3 – Perceptions of controlled human infection model studies for gonorrhoea

As discussed earlier, researchers at the University of Melbourne are proposing a controlled human infection model of throat gonorrhoea.

1. Can you tell me your initial reaction to the idea of this study of throat gonorrhoea being undertaken in Victoria?

2. Does it bring up any concerns for you?

3. What do you think the public health benefits might be?

4. What do you think the community reaction might be?

5. What challenges do you anticipate in the recruitment of participants and adherence to trial protocols for a gonorrhoea controlled human infection model of the throat?

6. Would your reaction be different if a gonorrhoea controlled human infection model of the penis study were being undertaken in Victoria?

7. Would your reaction be different if a controlled human infection model study for another infection, like malaria, typhoid or influenza were being undertaken in Victoria?

Part 4 – Opinions around the design and implementation of a gonorrhoea controlled human infection model

1. What would need to be in place for you to think an oropharyngeal gonorrhoea controlled human infection model could be implemented in Victoria?

2. What information do you think is important to include in information and consent materials to help potential participants to understand the risks and benefits of the study?

Final Comments

This brings us to the end of the set of questions for the interview.

2. Is there anything we have not covered today that you would like to comment on?

3. Are there any other experts that you recommend that we talk to?

End of Interview

That's all the questions we have for you today. I'm going to turn the audio recorder off to end the formal interview/focus group process. Thank you very much for volunteering your time today. The answers you provided will give us valuable insight into the acceptability of a controlled human infection model for gonorrhoea and will be important in guiding future research in this area. Would you like a summary of the study findings once it has finished? If yes, what email address should I send them too? Once again thank you so much for participating.

End the interview
